# Stability of Coumarins and Determination of the Net Iron Oxidation State of Iron–Coumarin Complexes: Implications for Examining Plant Iron Acquisition Mechanisms

**DOI:** 10.1021/acsearthspacechem.3c00199

**Published:** 2023-11-10

**Authors:** Kyounglim Kang, Walter D. C. Schenkeveld, Guenther Weber, Stephan M. Kraemer

**Affiliations:** † Environmental Geochemistry, Centre for Microbiology and Environmental Systems Science, University of Vienna, 1090 Vienna, Austria; ‡ Soil Chemistry and Chemical Soil Quality, Environmental Sciences, Wageningen University, 6708 PB, Wageningen 6700 AA, The Netherlands; § Leibniz-Institut für Analytische Wissenschaften − ISAS, 44227 Dortmund, Germany

**Keywords:** esculetin, scopoletin, fraxetin, phenolics, root exudate, Ferrozine, iron redox speciation, iron binding ligands

## Abstract

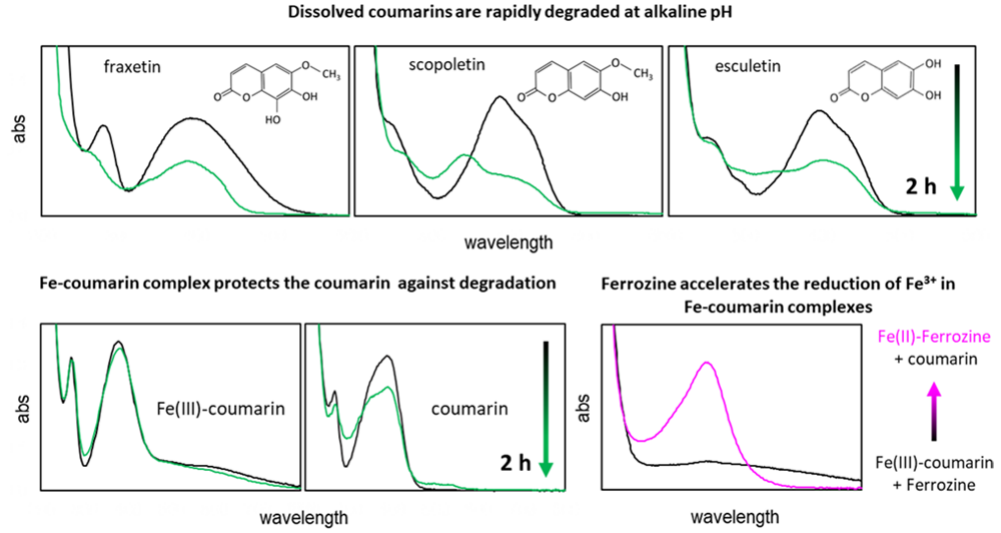

Coumarins are exuded into the soil environment by plant roots in response to iron (Fe) deficiency. Previous studies have shown that coumarins can increase the Fe solubility upon interaction with sparsely soluble Fe(III) (hydr)oxide. However, the chemical mechanisms of Fe(III) (hydr)oxide dissolution by coumarins remain unclear. The high redox instability of dissolved coumarins and the interference of coumarins in determining the Fe redox state hinder the quantitative and mechanistic investigation of coumarin-induced Fe mobilization. In this study, we investigated the oxidative stability of three coumarins that have been found in root exudates, esculetin, scopoletin, and fraxetin, over a broad pH range under oxic and anoxic conditions. Our results show that the oxidation of coumarins is irreversible under oxic conditions and that oxidative degradation rates increased with increasing pH under both oxic and anoxic conditions. However, the complexation of Fe protects coumarins from degradation in the circumneutral pH range even under oxic conditions. Furthermore, we observed that Ferrozine, which is commonly used for establishing Fe redox speciation, can facilitate the reduction of Fe(III) complexed by coumarins, even at circumneutral pH. Reduction rates increased with decreasing pH and were larger for fraxetin than for scopoletin and esculetin. Based on these observations, we optimized the Ferrozine method for determining the redox state of Fe complexed by coumarins. Understanding the stability of dissolved coumarins and using a precise analytical method to determine the redox state of Fe in the presence of coumarins are critical for investigating the mechanisms by which coumarins enhance the availability of Fe in the rhizosphere.

## Introduction

Coumarin and its derivatives (collectively referred to as coumarins) are secondary plant metabolites that are widely distributed in soil environments.^1,2^ Recent studies have reported that nongraminaceous species such as *Arabidopsis thaliana* exude hydroxylated coumarins into their rhizosphere in response to iron deficiency.^3−12^ Fe deficiency typically occurs in well-aerated alkaline and calcareous soils where the bioavailability of iron (Fe) is constrained by the low solubility of ferric hydroxide minerals.^13^ Due to their reducing and ligating properties, coumarins may increase Fe solubility in the soil via two chemical mechanisms: reduction of ferric iron (Fe(III)) to the much more soluble ferrous iron (Fe(II)) and formation of soluble complexes with Fe(III) and Fe(II).^8,11,14^

Root exudates of Fe deficient plants exuding coumarins typically contain mixtures of hydroxylated coumarin derivatives such as scopoletin, esculetin, and fraxetin.^4,11,15,16^ Results from our recent study suggest that redox reactions at Fe(III) (hydr)oxide mineral surfaces can induce the transformation of one coumarin (e.g., scopoletin) into a variety of others (e.g., esculetin and fraxetin as well as di- and trimers).^17^ Potentially, each of these coumarins may play an important role in biological Fe acquisition. Despite their potential importance, the reactivity of coumarins and the chemical mechanisms by which they enhance Fe availability have scarcely been studied, in part due to methodological and experimental challenges.

For instance, the limited water solubility of coumarins over most of the environmentally relevant pH range and the redox instability of their dissolved species hamper the quantitative and mechanistic investigation of coumarin-induced Fe mobilization, even in laboratory-based experiments. Already, for the preparation of coumarin stock solutions, these features can lead to complications like incomplete dissolution and contamination of the stock with oxidation products. Up until now, three methods have been reported to dissolve coumarins: dissolution in (1) a methanol–water mixture (30–80% v/v methanol),^8,18^ (2) boiling water,^19^ and (3) a slightly alkaline aqueous solution.^20^ Coumarins readily dissolve in methanol due to their hydrophobic aromatic ring structure. Dissolving hydrophobic organic compounds in a methanol–water mixture is the most widely used method for preparing stock solutions.^21^ However, methanol can impact the reactivity of coumarins, particularly at the mineral surfaces. For example, methanol in mixed solvents (methanol + water) can significantly influence the surface charge of (oxyhydr)oxide minerals like goethite (α-FeOOH) and hematite (α-Fe_2_O_3_), thus affecting adsorption of organic and inorganic ions onto such surfaces.^22,23^ Therefore, the application of methanol mixed solvents complicates a quantitative analysis and interpretation of chemical reactions between coumarins and Fe(III) (hydr)oxide minerals. In boiling water, coumarin dissolution was reported to be fast and complete,^19^ yet while cooling the solutions to 25 °C, amorphous coumarin reprecipitated, making this method unsuitable for preparing stock solutions to be used at room temperature.

Alternatively, solid coumarins can be dissolved in moderately alkaline solutions (pH 7.5–8.5), in which the coumarin’s phenolic OH groups (partially) deprotonate, resulting in enhanced water solubility.^24^ However, in the presence of oxygen, dissolved coumarins can undergo oxidative degradation, e.g., via hydroxylation, quinone formation, and/or dimerization reactions. Such reactions can be accelerated under alkaline conditions.^17^ At present, dissolution under alkaline conditions appears to be the only potentially suitable method for preparing coumarin stock solutions in experiments examining coumarin reactivity with environmental surfaces. Hence, an understanding of the chemical stability of dissolved coumarins as a function of pH is essential for controlling the quality of coumarin stocks but also for avoiding aging of experimental samples. Therefore, in this study, the degradation of three coumarins commonly found in plant exudates, fraxetin, scopoletin, and esculetin, is examined over a broad pH range (pH 4–12.5) under oxic and anoxic conditions.

Another experimental challenge concerns establishing the redox speciation of mobilized Fe. As coumarins can reduce and chelate Fe in the rhizosphere, both soluble Fe(II) and Fe(III)–coumarin complexes may form. To investigate the prevalent mechanism of Fe mobilization by coumarins, a method is needed for quantifying the concentrations of both Fe redox species in the presence of coumarins. It is key that the net Fe redox state remain unaffected throughout the analytical procedure. Ferrozine-based colorimetric assays have been widely used for determining Fe(II) concentrations over the last century. It has been demonstrated that sample properties like pH,^25^ temperature,^26^ and ionic strength^27^ and the presence of competitive ligands^28^ and organic matter^29,30^ can affect the accuracy of the analysis. For the analysis of Fe redox speciation in coumarin solutions, there are three specific concerns. First, solutions of coumarins and their Fe(III) complexes absorb photons over a broad range of the UV–vis spectrum. This may include the wavelength commonly used for the Ferrozine assay (i.e., 563 nm),^25,29^ potentially leading to interference and an overestimation of the Fe(II) concentration. Second, both coumarins and Ferrozine can reduce Fe(III).^31,32^ It is presently unknown if the addition of Ferrozine to solutions with a circumneutral pH containing Fe(III) and coumarins may shift the redox equilibrium to an extent that Fe(III) is reduced to Fe(II), also leading to an overestimation of the Fe(II) concentration. Third, although an excess of Ferrozine has been shown to recover Fe(II) from an array of environmental samples, there are no reports on the efficiency and kinetics of the ligand exchange reaction for Fe(II) from coumarins to Ferrozine. Therefore, it is unclear if the exchange reaction will be complete and if equilibrium is reached on a practical time scale.

Hence, in this study, we have investigated whether the classical Ferrozine assay is suitable for analyzing Fe redox speciation in coumarin solutions at circumneutral pH or if adaptations are required. Specifically, we examined if the ligand exchange reactions from Fe(II)–coumarin to Fe(II)–Ferrozine complexes are complete, if spectral interferences from Fe(III)–coumarin complexes may compromise quantification of Fe(II) concentrations by the Ferrozine assay, and if Fe(III) reduction occurred due to addition of Ferrozine to solutions containing Fe(III) and coumarins. Based on our findings, we suggest modification of the Ferrozine assay to improve the accuracy and precision in quantifying Fe(II) concentrations in samples containing coumarins or their Fe(III) chelate complexes.

## Materials and Methods

### Materials

Esculetin (6,7-dihydroxycoumarin, C_9_H_6_O_4_, >98%, Alfa Aesar), scopoletin (6-methylesculetin, C_10_H_8_O_4_, >99%, Sigma-Aldrich), fraxetin (7,8-dihydroxy-6-methoxycoumarin, C_10_H_8_O_5_, >98%, Sigma-Aldrich), iron(II) chloride tetrahydrate (FeCl_2_·4H_2_O, >99%, Sigma-Aldrich), iron(III) chloride hexahydrate (FeCl_3_·6H_2_O, >99%, Merck), Ferrozine (C_20_H_13_N_4_NaO_6_S_2_·*x*H_2_O, >98%, ACROS organics), ammonium acetate (CH_3_COONH_4_, >98%, Merck), sodium hydroxide (NaOH, >99%, Merck), hydrochloric acid (HCl, 30% supra pure grade, Merck), and sodium chloride (NaCl, >99.5%, Merck) were used as received without further purification. Piperazine-1,4-bis(propane-sulfonic acid) (PIPPS, C_10_H_22_N_2_O_6_S_2_, p*K*_a1_ = 3.73, p*K*_a2_ = 7.96, >97% pure, Merck), 2-morpholinoethanesulfonic acid monohydrate (MES, C_6_H_14_N_2_·H_2_O, p*K*_a_ = 6.06, >99%, Merck), 3-(*N*-morpholino)propanesulfonic acid (MOPS, C_7_H_15_NO_4_S, p*K*_a_ = 7.20, >99%, Carl Roth GmbH + Co), 1,4-dimethylpiperazine (DEPP, C_6_H_14_N_2_, p*K*_a1_ = 4.48, p*K*_a2_ = 8.58, >98% Sigma-Aldrich), and *N*,*N*,*N*′,*N*′-tetramethylethylenediamine (TEEN, (CH_3_)_2_NCH_2_CH_2_N(CH_3_)_2_, p*K*_a1_ = 6.58, p*K*_a2_ = 9.88, >99.5%, Sigma-Aldrich) were used as pH buffers. All experimental and analytical solutions were prepared with ultrapure water (resistivity > 18.2 MΩ·cm, TOC < 2 ppb, Milli-Q, Millipore).

### Preparation of Stock Solutions of Coumarins

To prevent oxidative degradation of the coumarins, stock solutions for fraxetin, scopoletin, and esculetin were prepared in an anaerobic chamber (mBRAUN, unilab 7185) under a N_2_ atmosphere at room temperature (20 ± 1 °C). A O_2_ concentration in the gas phase of less than 1 ppm was monitored and controlled. Water was preboiled for more than 1 h and was purged with N_2_ while cooling to 60 °C before introduction into the anaerobic chamber. For the introduction of N_2_-purged water and solid materials into the anaerobic chamber, the atmosphere in the antechamber was exchanged with N_2_ gas at least 10 times. The water and solid materials were stored overnight in the chamber prior to usage. 2.5 mM coumarin stock solutions were prepared by adding water to the solid coumarins and gradually increasing the solution pH to 8.1–8.3 through addition of 10–20 μL aliquots of 0.1 or 0.5 M NaOH solutions until a clear bright yellow solution was obtained. The pH of the solutions was carefully monitored with a pH meter (Orion 3 star, Thermo) throughout the dissolution process in order to prevent increasing the solution pH further than necessary for complete dissolution. The stock solutions were kept in aluminum foil wrapped 50 mL CELLSTAR Polypropylene Tubes (Cat. No.: 210261, Greiner) in the anaerobic chamber.

### Spectrophotometric Analysis of the Stability of Dissolved Coumarins and Fe–Coumarin Complexes

The chemical stability of coumarins and Fe–coumarin complexes was examined as a function of time at 20 ± 1 °C by UV–vis spectrophotometry (Varian Cary 50). Absorbance spectra were measured over a wavelength range from 200 to 800 nm. We focused our interpretation on the absorbance peak in the high UV–low vis range (maximum absorbance (λ_max_) between 340 and 430 nm).^8,33^ We specifically monitored the absorbance at λ_max_ of the original coumarin compound (i.e., λ_max_ at *t* = 0), which was coumarin and treatment specific. Changes over time in peak height and λ_max_ were interpreted as indicators for the degradation of the original coumarin compound. Identification of degradation products and determination of their specific absorbance fell outside the scope of this study. It should, however, be noted that degradation products may also absorb light in the aforementioned wavelength range. Therefore, interpretation of the remaining absorbance at λ_max_ of the original coumarin as a measure for its concentration may result in an overestimation; in fact, it represents the maximum possible remaining concentration (in case there is no interference from degradation products), corresponding to a lower limit for degradation.

### pH-Dependent Stability of Dissolved Coumarins

The effect of pH on the chemical stability was examined under oxic conditions (pO_2_: 0.2 atm) for the pH range of 4 to 12.5. Reactors (Polystyrene, Greiner) with experimental coumarin solutions containing 42 μM fraxetin, scopoletin, or esculetin and 0.01 M NaCl as background electrolyte were prepared under anaerobic conditions and taken from the anaerobic chamber just before starting the experiments. The pH was set by adding NaOH solution and buffered with 5 mM PIPPS (pH 4 and 8.5), MES (pH 6), MOPS (pH 7), DEPP (pH 9.5), or TEEN (pH 10.5). These buffers do not absorb light at wavelengths larger than 240 nm and do not complex trace metals like Fe.^34,35^ For the treatments at pH 11.5 and 12.5, no buffer was applied. The pH of the coumarin solutions was monitored and maintained throughout the experiments (ΔpH = ±0.05), using small aliquots of 0.01 and 0.05 M NaOH if necessary. In order to transfer oxygen efficiently, the reactors were purged with air by using a peristaltic pump (40 rpm). 0.1 μm PVDF filters (0.1 μm, Millipore, catalog no. SLVV033RS) were attached to the tubes connected to the pump to prevent dust particles from entering into the reactors. *t* = 0 corresponds to the moment of starting the air pump. Samples were drawn periodically during 24 h and were analyzed immediately.

Additionally, anaerobic control experiments were done at pH 10.5 and 12.5 in the anaerobic chamber (pO_2_ < 1 ppm (i.e., 10^–6^ atm), corresponding with an equilibrium O_2_ solution concentration of <13 nM; hence, coumarin oxidation by O_2_ can be assumed negligible under these conditions). The pH of the coumarin solutions was set directly before sampling started (*t* = 0). Samples were taken out of the anaerobic chamber immediately and analyzed on a UV–vis spectrophotometer within 1 min. The cuvette containing the sample was capped, and the headspace was minimized to minimize exposure to atmospheric oxygen during the measurement.

In order to examine the effect of temporarily overshooting the pH under oxic conditions on the stability of dissolved coumarins, experiments were conducted in which the pH of coumarin solutions was increased to 10.5 or 12.5 by adding NaOH, maintained at that pH for 2 h, and then decreased to pH 8.5 by adding HCl and PIPPS buffer. For these experiments, *t* = 0 corresponds to the moment when the pH was decreased to 8.5.

### Stability of Fe–Coumarin Complexes

The stability of Fe(II)– and Fe(III)–coumarin complexes was examined spectrophotometrically at circumneutral pH (pH 6 (MES), pH 7 (MOPS), and pH 8.5 (PIPPS)) under oxic and anoxic conditions. By mass spectroscopy, it is shown that Fe and coumarins form complexes in a 1:2 ratio at pH 6.5 and in a 1:3 ratio at pH 8.5 conditions (Figure S1). To ensure a stoichiometric excess of coumarins over Fe, they were mixed in a molar ratio larger than 3. Fe–coumarin solutions containing 42 or 83 μM fraxetin, scopoletin, or esculetin, 10 μM Fe(II) or Fe(III), 0.01 M NaCl, and 5 mM pH buffer were prepared under anaerobic conditions. For oxic treatments, the Fe–coumarin solutions were taken from the anaerobic chamber just before the experiments. For anoxic treatments, *t* = 0 corresponded to the moment of fixing the pH of the experimental solution, and for oxic treatments, it corresponded to the moment of starting the air pump (see previous section for aeration procedure). Samples from the Fe–coumarin solutions were taken periodically over 24 h and were immediately analyzed.

### Ferrozine Assay for Determining Fe(II) Concentrations in Solutions Containing Coumarins

Potential spectral interferences of Fe–coumarin complexes with the Ferrozine assay for determining Fe(II) concentrations were examined by adding Ferrozine to solutions containing Fe(II), Fe(III), Fe(II)–coumarin complexes, Fe(III)–coumarin complexes, and a mixture of both Fe(II)– and Fe(III)–coumarin complexes. For treatments involving Fe–coumarin complexes, Fe and coumarin stock solutions were mixed first in order to allow Fe–coumarin complexes to form (2 h), before addition of Ferrozine. The analyzed samples contained reactants in the following concentrations: 10 μM Fe(II), 10 μM Fe(III), 83 μM coumarin, 3 mM Ferrozine, 0.01 M NaCl, and 5 mM of the pH buffers (pH 6, 7, and 8.5). All experimental solutions and samples were prepared under anaerobic conditions. Samples were taken out of the anaerobic chamber just before analysis. The spectra of the samples were analyzed by UV–vis spectrophotometry over the wavelength range of 200 to 800 nm. Total Fe concentrations were measured by inductively coupled plasma mass spectrometry (ICP–MS, Agilent-7700; limit of quantification: 0.8 μg L^–1^ Fe (0.014 μM)).

Changes in Fe redox speciation due to reduction of Fe(III) in Fe(III)–coumarin complexes by Ferrozine were investigated under anoxic conditions at pH 6, 7, and 8.5. Absorbance was measured repeatedly for the wavelength range from 400 to 800 nm, and Fe(II) concentrations were determined using the Ferrozine assay (563 nm; ε = 2.86 × 10^4^ M^–1^ cm^–1^). Reduction rates were calculated from the slopes of linear regression lines of the Fe(II) concentration as a function of time; the time interval used for the regression was from 0 to 4 h (for treatments in which more than 50% of the Fe(III) was reduced within 4) or from 0 to 24 h (for treatments in which less than 50% of the Fe(III) was reduced within 24 h) (Figure S26).

### Spectrophotometric Analysis Dissolved Coumarins in Methanol–Water Mixed Solvent

The effects of solvents on the spectra of the dissolved coumarins were examined by dissolving coumarins in water and in methanol–water mixed solvent (5%/95% methanol–water mixture) under anoxic conditions at pH 6, 7, and 8.5. The coumarin solutions in methanol–water mixed solvent were prepared by dissolving coumarins in 99.8% methanol and adding water to make a final methanol concentration of 5%. The absorbances of coumarins in 5% methanol were measured 2 h after the mixing in the anaerobic chamber. The samples were taken out of the chamber just before analysis. The absorbances of the dissolved coumarins in methanol–water mixed solvent were measured by UV–vis spectrophotometry over the wavelength range from 200 to 800 nm and compared with the absorbances of the dissolved coumarins in water.

## Results

### Stability of Hydroxylated Coumarins as a Function of pH

UV–vis absorbance spectra were measured in solutions with 42 μM coumarin under atmospheric conditions over a pH range from 4 to 12.5 (Figure S2a for fraxetin, Figure S3a for scopoletin, and Figure S4a for esculetin). The precipitation of oxidation products was not observed during the experiments for any of the examined coumarins. The spectra for the three coumarins had similar features: with increasing wavelength, absorbance first declined, reaching a local minimum in the range of 260 to 325 nm, followed by a characteristic absorbance band extending into the visible wavelengths with a local maximum between 330 and 400 nm (λ_max_). This band corresponds with the keto group in the α-pyrone ring.^36^ Depending on pH and coumarin, molar extinction coefficients (ε) for this band at λ_max_ ranged from 330 to 400 nm (Table S1); ε was consistently the smallest for fraxetin. Additional smaller, pH-dependent features for fraxetin (pH 7–12.5; λ_max_ = 265–275 nm), scopoletin (pH 4–7; λ_max_ = 285–295 nm), and esculetin (pH 4–7; λ_max_ = 305 nm; pH 4–7; λ_max_ = 250 nm) were also observed. The spectra of the three coumarins showed a bathochromic shift of 40 to 60 nm for the largest local absorbance maximum, occurring over the pH range from 6 to 8.5, presumably corresponding to deprotonation of the first phenolic OH group (p*K*_a_: 7.6 for esculetin,^37^ 7.9 for fraxetin,^3^ and 8.3 for scopoletin^33^). For scopoletin and esculetin, the shift in λ_max_ coincided with an increase in ε by a factor of approximately 1.5. Between pH 10.5 and 12.5, a second shift and a broadening of the absorbance band toward larger wavelengths occurred. For fraxetin and esculetin, this may be related to deprotonation of the second phenolic OH group; for scopoletin, oxidative demethylation might occur.

**Figure 1:**
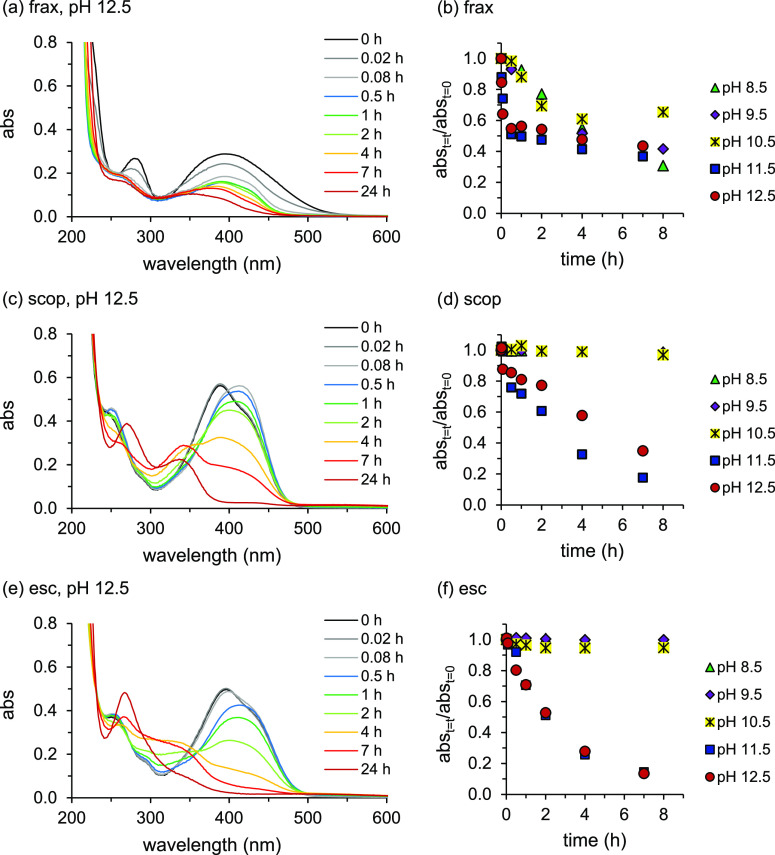
Change in UV–visible absorbance (abs) spectra over time under oxic conditions at pH 12.5 is shown for (a) fraxetin (frax), (c) scopoletin (scop), and (e) esculetin (esc) (initial concentration: 42 μM). The decreasing absorbance at the initial λ_max_ around 390 nm as a function of time at various pH values under oxic conditions is shown in (b) frax (λ_max_: 393 nm), (d) scop (λ_max_: 389 nm), and (f) esc (λ_max_: 396 nm) (full spectra can be seen in Figures S2, S3, and S4).

Among the three coumarins, fraxetin had the poorest stability under alkaline pH conditions. As shown in Figure 1a, the fraxetin spectra are characterized by two absorbance bands between 200 and 600 nm; one representing the keto group in the α-pyrone ring (λ_max_ = 390 nm) and one representing deprotonated 7- and 8-OH groups in the catechol structure (λ_max_ = 286 nm).^38^ Upon oxidation, the band intensity at both wavelengths decreased. The absorbance peak at 286 nm rapidly disappeared within 0.08 h, while the local maximum absorbance peak around 390 nm decreased more gradually over 24 h, with λ_max_ shifting toward smaller wavelengths. This implies that at pH 12.5 the keto group in the α-pyrone ring is relatively stable against oxidation in comparison to the catechol hydroxyl groups.

The rate of fraxetin degradation, represented by the decline in absorbance at the initial λ_max_ = 390 nm, was strongly pH dependent (Figure 1b). At pH 11.5 and 12.5, two stages were distinguished: a fast initial degradation of 45–50% during the first 0.5 h and a slower decline afterward. Our data can neither provide insight into the oxidation mechanism nor elucidate the observed two stages. Possibly, keto groups containing oxidation products formed during the first 0.5 h have a higher redox potential preventing further oxidation, or oxidation products may contain newly formed moieties that absorb light at wavelengths near 390 nm. For the pH range of 8.5–10.5, initial degradation progressed much slower, at a comparable rate for this complete pH range (Table S2), and was approximately linear with time up until 4 h. For the pH range of 4–7, no significant spectral changes (i.e., no indications for degradation) were observed during 8 h, which is consistent with our previous observations.^17^

Scopoletin proved considerably less susceptible to oxidative degradation than fraxetin; no significant spectral changes were observed during 24 h for pH values up to 10.5 (Figures 1d and S3), and initially, changes in absorbance at λ_max_ developed more slowly for the pH range 11.5–12.5 (Figure 1c,d). At pH 12.5, the scopoletin spectra showed a single band in the wavelength range from 200 to 600 nm, corresponding with the keto group in its α-pyrone ring (Figure 1c). During the first 0.08 h of exposing scopoletin to oxygen, λ_max_ shifted from 386 to 413 nm, which is consistent with observations on scopoletin oxidation by Horseradish peroxidase.^39^ Between 0.02 and 1 h, a new absorbance peak appeared near 250 nm, suggesting the formation of a catechol structure via oxidative demethylation and generation of a 6-OH group on the benzene ring.^17,40^ However, upon further oxidation, absorbance at 250 nm declined and the peak transformed into a shoulder at around 250–270 nm. Furthermore, after 2 h, a broad new absorbance band emerged between 250 and 500 nm (λ_max_ ≈ 350 nm) replacing the band with λ_max_ = 413 nm.

For evaluating the degradation of scopoletin at pH 12.5, absorbance at the initial λ_max_ = 386 nm was assessed. As Figure 1d shows, absorbance declined in 2 stages: a fast initial stage up to 0.08 h related to the aforementioned peak shift, followed by a gradual linear decline over time. This linear decline was somewhat faster at pH 11.5 than that at pH 12.5. It cannot be excluded that, already after the initial peak shift, no scopoletin was left.

Esculetin showed a susceptibility to oxidative degradation similar to that of scopoletin with no significant spectral changes for pH values up to 10.5 and a gradual decline in absorbance at λ_max_ for pH 11.5–12.5. At pH 12.5, the esculetin spectrum had one absorbance band (λ_max_ = 395 nm) corresponding to the keto group and one shoulder (∼260 nm). Similar to scopoletin, a shift in λ_max_ was observed, from 395 to 413 nm between 0.08 and 0.5 h. Throughout the experiment, the shoulder at around 260 nm disappeared and a new absorbance peak at 270 nm appeared.

For evaluating the degradation of esculetin at pH 12.5, absorbance at the initial λ_max_ = 395 nm was assessed. As shown in Figure 1f, the absorbance gradually decreased over time, at a comparable rate for pH 11.5 and 12.5, somewhat faster than for scopoletin.

In general, the observed increased susceptibility to oxidation with increasing pH results at least in part from the simultaneous decrease in the reduction potential of the examined coumarins, which is directly related to the pH-induced deprotonation of hydroxyl groups. Furthermore, the substitution of a hydroxyl group by a methoxy group decreases the rate of oxidation.^41^

In a previous study, we had observed by cyclic voltammetry that fraxetin was more prone to oxidize already at lower pH values than scopoletin and esculetin,^17^ in line with our current findings.

Results from an additional experiment with higher coumarin concentrations (2.5 mM) indicate that the rate at which the spectrum changes increases with the concentration of the coumarin solution (Figure S5). This suggests that degradation may be enhanced at higher concentrations, possibly because dimerization/oligomerization reactions with a reaction order larger than 1 are facilitated.

### Reversibility and the Role of Oxygen in pH-Induced Structural Changes in Coumarins

The results from the experiment on pH-dependent stability of coumarins raised two further questions: “Are changes in the coumarin structure due to (temporarily) elevated pH values (>10) reversible?” and “Does pH-induced decomposition of coumarins occur even in the absence of oxygen?” In a practical context, answering the first question is of particular importance for deciding whether or not to continue or start anew when preparing a coumarin stock solution and accidentally excessively raising the pH; can a pure stock solution still be obtained by lowering the pH again, or will the impurities produced at elevated pH persist? Answering the second question will provide insight into what extent coumarin degradation can be prevented by preparing and preserving stock solution under anaerobic conditions.

**Figure 2:**
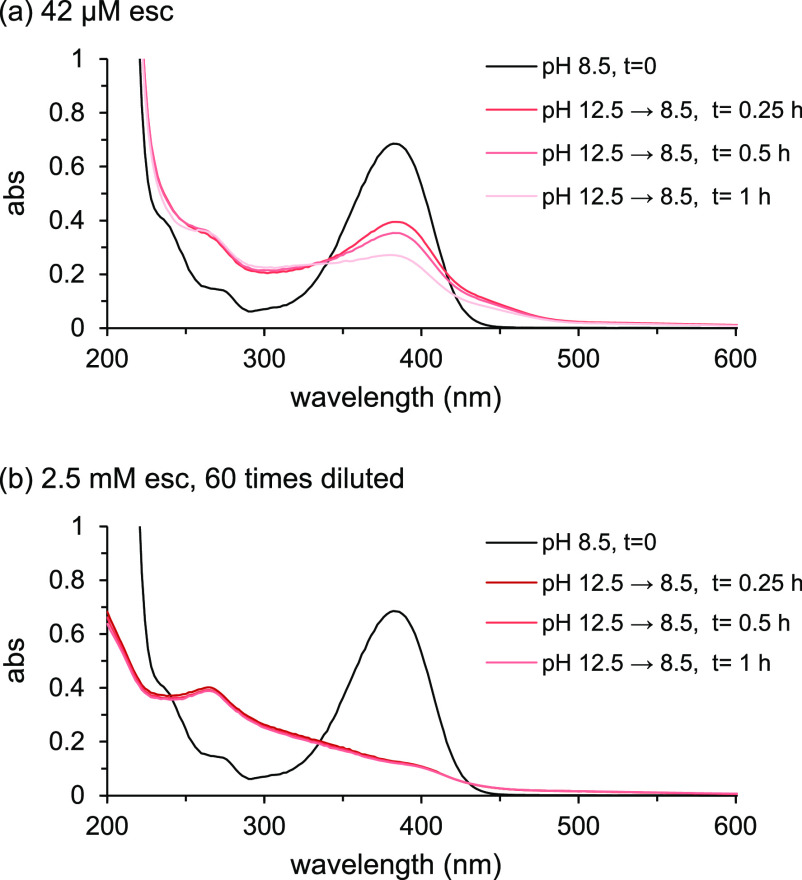
Changes in UV–visible absorbance (abs) spectra of esc over time at pH 8.5 under oxic conditions (initial esc concentration: (a) 42 μM and (b) 2.5 mM). For the red spectra, the solution pH had first been increased to 12.5 and had been maintained at this level for 2 h before setting it to 8.5, all under oxic conditions; *t* = 0 corresponds to the moment the pH was set to 8.5 (first measurement time after 0.25 h). The results of control treatments (pH had not been increased) for pH 8.5 are presented as black spectra. For the 2.5 mM esc treatment (b), samples were diluted 60 times in order to examine the absorbance (<1) between 200 and 600 nm.

In order to answer the first question, the pH of 42 μM and 2.5 mM coumarin solutions was raised to 10.5 and 12.5, maintained at that level for 2 h, and then lowered to pH 8.5 in a setup shown in Figure S6, all under oxic conditions. The reversibility of changes to the coumarin structures induced at high pH and the stability of coumarin solutions after lowering the pH to 8.5 were assessed by (changes in) the UV–vis spectra, starting from the moment the pH was set to 8.5. For esculetin solutions for which the pH had been raised to 12.5, results are presented in Figure 2. Corresponding results for fraxetin and scopoletin are presented in Figure S7.

After the pH was adjusted to 8.5, following the temporary rise to 12.5, absorbance at the initial λ_max_ = 384 nm remained considerably lower than for a control at pH 8.5 that had not undergone the pH shift. This implies that degradation reactions at higher pH were irreversible. For the 42 μM treatment that had undergone the pH shift, the decline in absorbance after 0.25 h at pH 8.5 relative to the control (42%) (Figure 2a) was comparable to the decline in absorbance after 2 h at pH 12.5 (47%) (Figure 1f). Interestingly, after the solution pH was lowered to 8.5, the absorbance at λ_max_ continued to decline, suggesting that degradation of esculetin proceeded at a pH value where no apparent degradation occurred when starting with a “fresh” solution (Figure S3). For the more concentrated 2.5 mM esculetin solution (Figure 2b), degradation at pH 12.5 was faster, up to 83%, and did not continue once the pH was set to 8.5; as suggested, possibly, the faster degradation is because the dimerization/oligomerization rate increased with concentration. Also, fraxetin and scopoletin degradation at pH 12.5 proved to be irreversible (Figure S7). Contrary to esculetin and scopoletin, for fraxetin, the absorbance at λ_max_ in the 42 μM treatment did not further decrease after the pH was set to 8.5. For the treatments in which the pH had been temporarily raised to 10.5, a decline in absorbance at λ_max_ ≈ 390 nm relative to the control was observed for all coumarins after the pH was lowered to 8.5 (Figure S8). Especially for scopoletin and esculetin, this is surprising, as in the constant pH treatments neither at pH 10.5 nor at pH 8.5, changes in the spectra this pronounced had been observed (Figures S3 and S4). Possibly, the coumarins underwent changes at pH 10.5 that are not visible in the UV–vis spectra, but that triggered a further reaction when the pH is lowered to 8.5 that does lead to changes in the spectrum.

**Figure 3:**
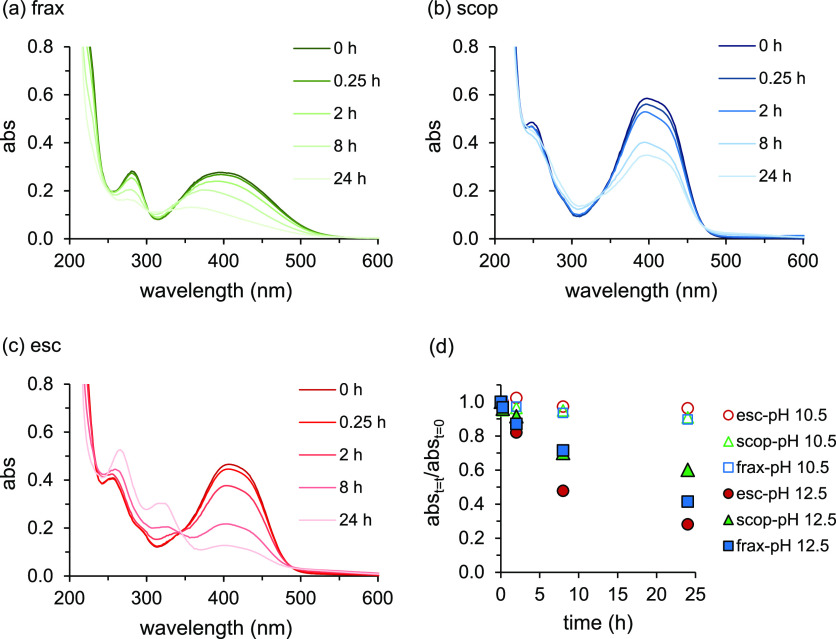
Change in UV–visible absorbance (abs) spectra over time at pH 12.5 under anoxic conditions of (a) frax, (b) scop, and (c) esc (initial concentration: 42 μM). (d) Decrease in absorbance at the initial λ_max_ around 390 nm of the coumarins as a function of time at pH 10.5 (spectra in Figure S9) and 12.5 under anoxic conditions.

Also under anoxic conditions, a decline in absorbance at λ_max_ ≈ 390 nm was observed at pH 12.5 in 42 μM solutions of all three coumarins (Figure 3a–c). This implies that the presence of oxygen is not required for coumarins to degrade. Degradation at pH 12.5 did, however, go slower under anoxic conditions (Figure 3d) than under oxic conditions (Figure 1b,d,f). Contrary to that under oxic conditions, the bathochromic shift within the first 0.5 h was not observed for scopoletin and esculetin at pH 12.5 under anoxic conditions. This implies that degradation reactions followed different pathways under oxic and anoxic conditions and that the shift occurred due to oxidation. Because of the lack of the bathochromic shift, the fast initial decline in absorbance at λ_max_ observed for esculetin and scopoletin under oxic conditions was also missing. Small changes in absorbance at λ_max_ were also observed at pH 10.5 (Figure S9), but the decrease in absorbance at λ_max_ did not exceed 10% (Figure 3d). For scopoletin and esculetin, these changes were comparable to the changes under oxic conditions (Figure 1d,f); for fraxetin, changes under oxic conditions were much larger (Figure 1b). Also for anoxic conditions, the reversibility of spectral changes induced at pH 12.5 in 2.5 mM coumarin solutions was examined after lowering the pH to 8.5. Under anoxic conditions, changes proved almost entirely reversible: the spectra for the coumarin solutions 1 h after the pH had been lowered to 8.5 was almost identical with the spectrum (>240 nm) of “fresh” coumarin solution at pH 8.5 (Figure S10).

Additionally, spectra were compared for coumarins dissolved in methanol, which was subsequently blended with water to a 5%/95% methanol–water mixture, and coumarins dissolved in water (Figure S11). The coumarin spectra for both solvents are similarly shaped with the methanol–water spectra having a 3–15% larger absorbance at λ_max_ for the keto group, depending on the coumarin and the solution pH. Because, during dissolution in water under anoxic conditions, the pH had been increased only to 8.3, well below the threshold above which spectral changes had been observed for scopoletin and esculetin, solvent effects seem a more likely cause for differences between the spectra than degradation during preparation.

### Fe–Coumarin Spectra

**Figure 4:**
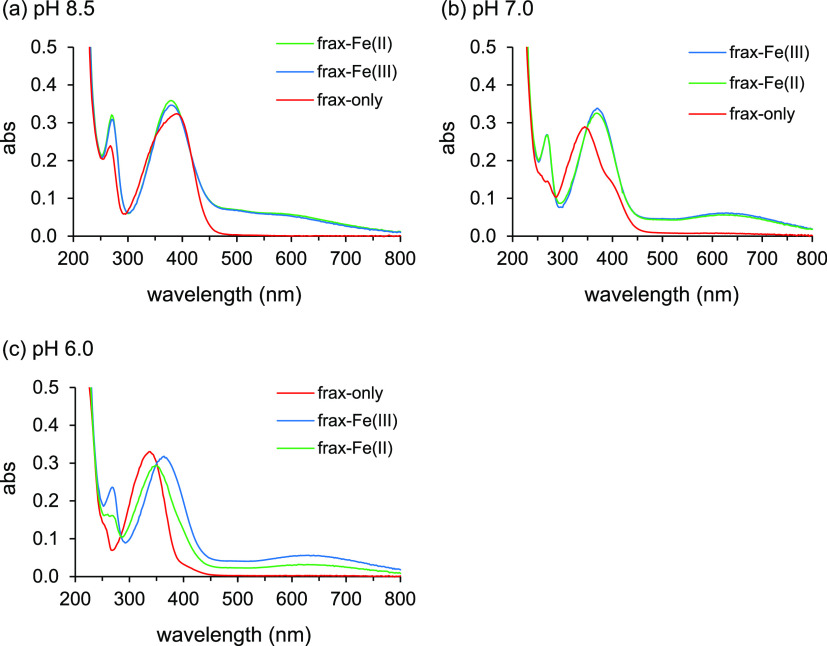
UV–visible absorbance (abs) spectra of fraxetin (frax, initial concentration: 42 μM) in the presence and absence of 10 μM Fe(II) or Fe(III) at (a) pH 8.5, (b) pH 7.0, and (c) pH 6.0 under anoxic conditions (*t* = 0 h: samples were analyzed immediately after taking the sample out of the glovebox).

The effect of Fe(II) and Fe(III) addition to coumarin solutions on the UV–vis absorbance spectra was examined for the pH range of 6–8.5. The coumarin and Fe concentrations were set to 42 and 10 μM, respectively. To prevent precipitation of Fe(hydr)oxide minerals, the Fe to ligand ratio was smaller than 1:3, providing an excess of free ligand over the entire examined pH range (Figures 4, S13, and 14). The spectra were measured immediately after taking the sample out of the glovebox (within 2 h after Fe addition (*t* = 0)).

At pH 6, Fe(III) addition to fraxetin caused a bathochromic shift in the peak related to the keto group, the emergence of a new peak at around 265 nm, and the development of a broad peak in the 450–800 nm range (Figure 4c). The first two effects were also observed in relation to an increase in pH from 6 to 8.5 and are presumably (partly) resulting from deprotonation of the catechol groups due to complexation of Fe(III). The broad peak in the visible wavelength range upon formation of Fe(III) complexes represents the ligand-to-metal charge transfer band, typically observed for coumarins and other Fe–catecholate complexes.^8,42,43^ Fe(II) addition had similar effects on the spectrum as Fe(III) addition was less pronounced; the bathochromic shift was smaller and the broad peak less high, suggesting fraxetin was complexed to Fe(II) to a smaller extent than to Fe(III). At higher pH values (7–8.5), the spectra for Fe(II) and Fe(III) fraxetin solutions were almost identical, implying that very similar complexes had formed (Figure 4a,b). With the exception of the broad peak/tailing (450–800 nm), at pH 8.5, the spectra of the fraxetin solutions with and without Fe addition were very similar, as deprotonation also occurred in the absence of Fe.

For scopoletin and esculetin, our findings were similar to those of fraxetin (Figures S13 and S14); the peak at 265–275 nm was, however, less pronounced (esculetin) or missing (scopoletin), and at pH 6, the spectra of the free ligand and the ligand with Fe(II) were almost identical. Our data suggest that at pH 6 Fe(II) forms complexes with coumarins to a much lesser extent than Fe(III), probably because Fe(III) more effectively competes with protons for binding to the catecholate group due to its larger charge-to-radius ratio.

Although the formation of Fe scopoletin complexes appears counterintuitive because scopoletin lacks a catechol group, 1:2 and 1:3 complexes have been found by mass spectroscopy.^8,17^ A direct comparison between our UV–vis absorbance spectra of Fe coumarin complexes and those reported in Schmidt et al.^8^ is not possible, since they were recorded in acetonitrile as solvent with added trimethylamine.

### Stability of Coumarins in the Presence of Fe

Formation of organometal complexes can prevent degradation of ligands, potentially prolonging their residence time in the environment and can stabilize the redox state of the complexed metals.^44,45^ In order to assess such effects for coumarins, we investigated coumarin degradation in the presence of Fe(II) or Fe(III) at pH 6–8.5 under oxic and anoxic conditions by examining the change in absorbance spectra of the Fe coumarin complexes (vide supra) over time.

**Figure 5:**
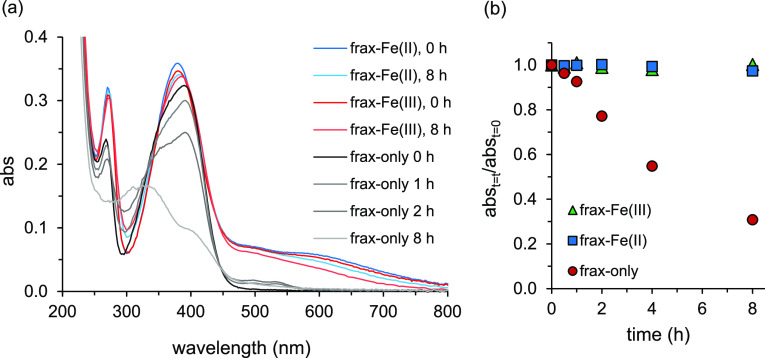
UV–visible absorbance (abs) spectra of fraxetin (initial concentration: 42 μM) in the presence and absence of 10 μM Fe(II) or Fe(III) at pH 8.5 under oxic conditions as a function of time. Full spectra between 250 and 800 nm (a) and absorption at the absorption maximum at *t* = 0 (390 nm) (b).

In Figure 5, results are presented for fraxetin at pH 8.5 under oxic conditions; fraxetin is of particular interest as the free ligand was shown to degrade under oxic conditions at (environmentally relevant) circumneutral pH (>7; Figure S2). Results for all three coumarins with Fe(II) and Fe(III) at pH 6, 7, and 8.5 are presented in Figures S15–S20.

The fraxetin degradation rate, as assessed by the decline in absorbance at λ_max_ ≈ 390 nm, was considerably smaller in solutions to which Fe had been added (up to 5% after 8 h) than in the solution to which no Fe had been added (70% after 8 h) (Figure 5b). The resistance against degradation resulting from the complexation of Fe(II) and Fe(III) is comparable. The degradation rate is not linearly related to the excess (i.e., non-Fe-complexed) ligand concentration: the treatment with only free ligand had ∼4 times more free ligand, but the degradation rate was 14 times faster. Possibly, this is related to the nonlinear effect of the free coumarin ligand concentration on the rate of degradation reactions mentioned before.

In the presence of Fe(II), the degradation of coumarins under oxic conditions (Figures S15–S17) was comparable to or smaller than for the ligand-only at pH 7 and 8.5 (Figures S2–S4). At pH 6, particularly for scopoletin and esculetin, the spectra showed a gradual emergence of the shoulder in the 450–800 nm range observed for Fe(III) complexes. A gradual oxidation of uncomplexed Fe(II) to Fe(III) and subsequent formation of Fe(III) complexes seems the most probably explanation. Yet, slow formation of Fe(II) complexes with a very similar spectrum as Fe(III) complexes (as shown for pH 8.5, Figures 4, S13, and 14) cannot be excluded.

In the presence of Fe(III), at pH 8.5, the coumarin spectra hardly changed over time, with the exception of fraxetin under oxic conditions. For esculetin and fraxetin, contrary to scopoletin, changes were observed at lower pH, typically more pronounced under oxic conditions: the tailing diminished, and the absorbance band related to the keto group underwent a hypsochromic shift (Figures S18 and S20). This suggests Fe(III) complex dissociation, leading to formation of the free ligand.

### The Ferrozine Assay: Fe(II) Recovery and Spectral Interference by Fe(III)–Coumarin Complexes

**Figure 6:**
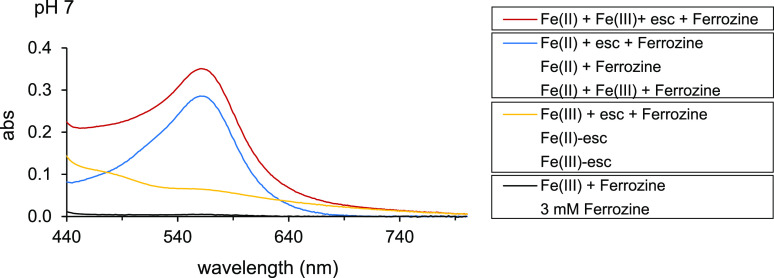
UV–visible absorption (abs) spectra of Ferrozine-only and Ferrozine in the presence of Fe(II) and/or Fe(III) and/or esc at pH 7 under anoxic conditions (10 μM Fe(II), 10 μM Fe(III), 83 μM esc, and 3 mM Ferrozine). Fe and coumarin were mixed for 20 min before Ferrozine was added. After the addition of Ferrozine, the spectra were analyzed immediately. In the legend, treatments with spectra that strongly overlapped in the 440–800 nm range have been combined in a box. The individual overlapped spectra are presented in Figure S21.

In order to investigate if Ferrozine can effectively scavenge all Fe(II) from Fe(II)–coumarin complexes and if the spectra of Fe(III)–coumarin and Fe(II)–Ferrozine complexes interfere at the wavelength used for quantification of the Fe(II) concentration, we compared the spectra of solutions containing Ferrozine-only and Ferrozine in the presence of Fe(II) and/or Fe(III) and/or coumarins at pH 6–8.5. For esculetin at pH 7, spectra (440–800 nm) are presented in Figure 6; extended spectra (200–800 nm) and spectra for the remaining combinations of coumarins and pH values are presented in Figure S21.

At pH 7, Ferrozine-only showed no absorbance between 440 and 800 nm (including 563 nm used in the Ferrozine assay), and the presence of Fe(III) has no effect on the spectrum (Figure 6). The spectra for Fe(II)+Ferrozine and Fe(II)+esculetin+Ferrozine were similar to a broad absorbance band at 440–700 nm and the characteristic λ_max_ = 563 nm for Fe(II)–Ferrozine complexes while the spectra for Fe(II)–esculetin complexes showed the characteristic λ_max_ = 367 nm (Figure S21) and tailing up to 800 nm. This indicates that esculetin in Fe(II)–esculetin complexes was rapidly and completely displaced by Ferrozine to form Fe(II)–Ferrozine complexes. For other pH values and other coumarins, the addition of Ferrozine also resulted in the quantitative complexation of Fe(II) (Figure S21). This implies that Ferrozine is an effective scavenger for Fe(II) complexed by coumarins. At pH 7, the spectra for Fe(III)–esculetin in the presence and absence of Ferrozine were identical, with the typical tailing from 440 to 800 nm (Figure 6). This implies that, under these conditions, Ferrozine had not reacted with Fe(III) from Fe(III)–esculetin and indicates that Fe(III) had not been reduced during complexation. However, the reactivity of Ferrozine with Fe(III)–coumarin complexes proved strongly dependent on the coumarin and the pH (Figures S21 and 7) and will be discussed in more detail in the following section on Ferrozine-induced reduction of Fe(III) in Fe(III)–coumarin complexes.

**Figure 7:**
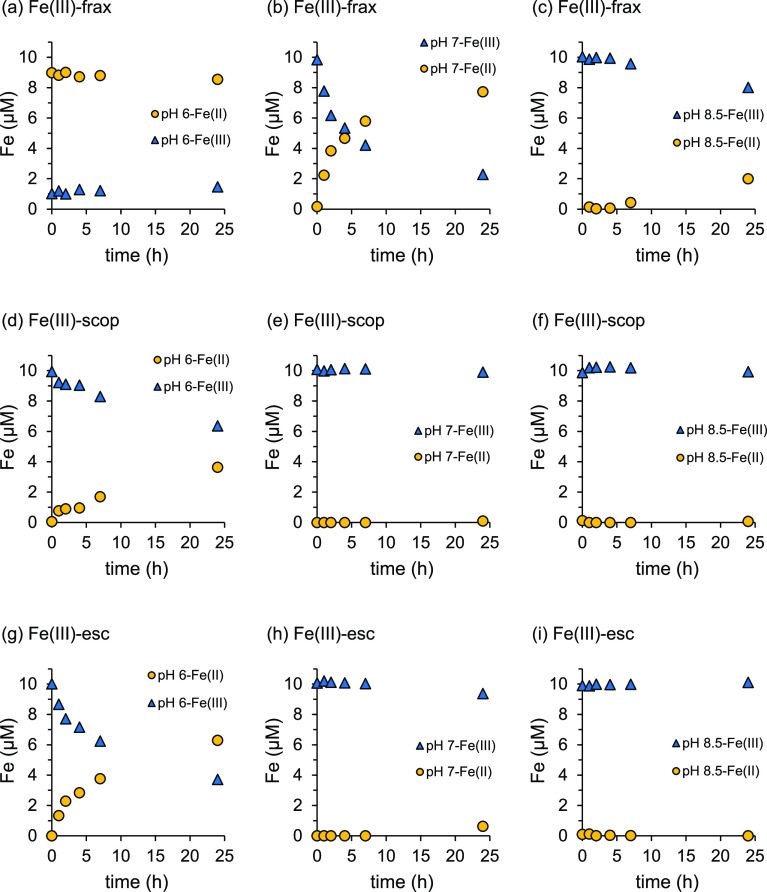
Changes in Fe redox speciation as a function of time in solutions containing (a–c) Fe(III)–frax, (d–f) Fe(III)–scop, and (g–i) Fe(III)–esc (10 μM Fe(III) and 83 μM coumarin) and 3 mM Ferrozine at pH 6, 7, and 8.5 under anoxic conditions.

The spectrum of the Fe(III)+Fe(II)+esculetin+Ferrozine treatment corresponds to the summation of the spectra for Fe(III)–esculetin and Fe(II)–Ferrozine. For the examined pH range of 6–8.5, all Fe(III)–coumarin complexes have absorbance at 563 nm (Figure S21). Therefore, in mixtures of Fe(II)– and Fe(III)–coumarin complexes to which Ferrozine is applied, the absorbance at 563 nm is not directly proportional to the Fe(II) concentration. The spectral interference of Fe(III)–coumarin complexes inhibits a direct quantification of Fe(II) concentrations by means of the Ferrozine assay as reported.^29,32^ For a stoichiometric excess of coumarin ligand, absorbance and hence the extent of interference increase linearly with the complexed Fe(III) concentration (Figure S22).

Because, upon Ferrozine addition, all Fe(II) will form Fe(II)–Ferrozine complexes and Fe(III) does not form complexes with Ferrozine, absorbance at 563 nm in solutions containing Fe(II), Fe(III), an excess of coumarin ligand, and Ferrozine can be described by
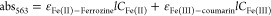
1where abs_563_ is the measured absorbance at 563 nm, ε_Fe(II)–Ferrozine_ and ε_Fe(III)–coumarin_ are the molar absorptivity coefficients for Fe(II)–Ferrozine and Fe(III)–coumarin, respectively, *l* is the optic path length (1 cm), and *C*_Fe(II)_ and *C*_Fe(III)_ are the Fe(II) and Fe(III) concentrations, respectively. Molar absorptivity coefficients at 563 nm of all Fe(III)–coumarin complexes and Fe(II)–Ferrozine were determined for pH 6–8.5 (Table 1). Furthermore, the total Fe solution concentration (*C*_Fe(tot)_), measured by ICP-MS, equals the sum of the Fe(II) and Fe(III) concentrations (*C*_Fe(II)_ and *C*_Fe(III)_, respectively):
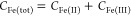
2Combining eqs 1 and 2 provides the following expression for the Fe(III) concentration as a function of the absorbance at 563 nm and the total Fe concentration:
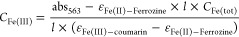
3*C*_Fe(II)_ can be calculated by filling *C*_Fe(III)_ into eq 2. In this approach, it is assumed that Fe does not form complexes with coumarin oxidation products.

**Table 1:** Molar Absorption Coefficients (M^–1^ cm^–1^) of Fe(II)–Ferrozine and Fe(III)–Coumarin Complexes at 563 nm

	Fe(II)–Ferrozine (M^–1^ cm^–1^)	Fe(III)–frax(M^–1^ cm^–1^)	Fe(III)–scop, M^–1^ cm^–1^	Fe(III)–esc (M^–1^ cm^–1^)
pH 6	28 600	4590	5950	6290
pH 7	28 600	4700	6300	6560
pH 8.5	28 600	6320	6730	6900

### Ferrozine-Induced Reduction of Fe(III) in Fe(III)–Coumarin Complexes

The procedure for determining the Fe(II) and Fe(III) concentrations described above does not take into account that Ferrozine may potentially affect the redox speciation by reducing Fe(III), leading to an overestimation of the Fe(II) concentration. The rate of Fe(III) reduction by Ferrozine may constrain both if and how the Ferrozine assay can be applied for determining Fe redox speciation in solution containing Fe–coumarin complexes: Fe redox speciation needs to be established before Ferrozine-induced Fe(III) reduction has a significant impact on it.

To examine for which coumarins and under what conditions the Ferrozine assay can be applied, we investigated the reduction rates of Fe(III) in Fe(III)–coumarin complexes in the presence of Ferrozine under anoxic conditions for pH 6–8.5. The initial concentrations of Fe(III), the coumarin, and Ferrozine were set to 10 μM, 83 μM, and 3 mM, respectively. In Figure 7, the Fe(II) and Fe(III) concentrations, calculated using eqs 2 and 3, are presented as a function of time; the underlying absorbance spectra are included in Figure S23, and the calculated initial Fe(III) reduction rates are presented in Table S3. Our results show that, in the absence of Ferrozine, Fe(III)–coumarin complexes are stable up to 8 h at pH 6 and up to 24 h at pH 7 and 8.5 (Figures S18–S20).

The Ferrozine-induced Fe(III) reduction rate strongly depended on the coumarin (fraxetin > esculetin > scopoletin) and increased with decreasing solution pH (6 > 7 > 8.5 for fraxetin and 6 > 7 ≈ 8.5 for scopoletin and esculetin) (Figure 6). The Fe(III) reduction rate corresponded to the stability of the coumarins against oxidation (Figures S2–S4). At pH 6, reduction of Fe(III) in Fe(III)–fraxetin was nearly complete almost instantaneously, and hence, no exact reduction rate could be determined. Instead, a minimum rate was estimated based on the change in Fe(III) concentration and the time between Ferrozine addition and analysis (1 min). For fraxetin, the Fe(III) reduction rate increased by more than 3 orders of magnitude between pH 6 and 8.5 (Table S3). At pH 6, the Ferrozine-induced Fe(III) reduction rates for Fe(III)–scopoletin (0.14 μM h^–1^) and Fe(III)–esculetin (0.68 μM h^–1^) were over 3 orders of magnitude smaller than for fraxetin. At pH 7 and 8.5, Fe(III) reduction for both Fe(III)–scopoletin and Fe(III)–esculetin during 24 h was small to negligible (<0.03 μM h^–1^). This aligns with the lack of reactivity of Ferrozine toward solid Fe(III) phases like lepidocrocite^46^ and ferrihydrite^47^ at circumneutral pH conditions.

Including Fe(II) in the Fe(III)–coumarin + Ferrozine mixture appeared to somewhat lower Ferrozine-induced Fe(III) reduction rates (Figure S24 and Table S3). Remarkably, at pH 8.5 for fraxetin and scopoletin, the Fe(III) concentrations gradually increased over 24 h. This suggests the oxidation of Fe(II) from Fe(II)–Ferrozine complexes, resulting in the formation of Fe(III)–coumarin complexes. It is unclear what the oxidant is as the experiment was carried out under anaerobic conditions. As the oxidation reaction was not observed for esculetin, possibly the methoxy group in scopoletin and fraxetin was involved.

The results presented above confirm that Ferrozine addition does affect Fe redox speciation in solutions containing coumarins. To accurately quantify the redox speciation, it is advisable to analyze samples shortly after Ferrozine addition, especially for fraxetin and for pH values below pH 7. Our results suggest that, for most combinations of coumarins and pH, spectroscopic analysis within 3 min after Ferrozine addition should not lead to changes in Fe(III) concentration larger than 1%. However, for Fe(III)–fraxetin at pH 6, Ferrozine-induced Fe(III) reduction was so fast that it is practically not feasible to accurately establish Fe redox speciation.

## Discussion

### Relation between the Stability against Degradation of Hydroxylated Coumarins and Their Structure

The stability of dissolved coumarins strongly depends on the substituents on their aromatic rings. Both the number and type of moieties (e.g., catechol and methoxy groups) and their position on the aromatic ring may affect the redox stability of the coumarin. The comparatively poor stability of fraxetin (7,8-OH, 6-OCH_3_) relative to scopoletin (7-OH, 6-OCH_3_) and esculetin (6,7-OH) may be related to the larger number of substituents on its aromatic ring or to specific interactive effects between the methoxy and the catechol group. In our recent study, we identified four main pathways for coumarin oxidation, leading to formation of dimers, quinones, demethylated coumarins, and (further) hydroxylated coumarins.^17^ Among the examined coumarins, only fraxetin is directly susceptible to all four pathways. The redox reactivity and oxidation pathways of catechol groups^38,48,49^ and the accelerating effect of strongly alkaline media^50^ have been extensively studied. A recent study reported that a methoxy group in phenolic structures like fraxetin and scopoletin can enhance the molecules redox reactivity by increasing the electron donation ability of the molecule.^51^ Upon oxidative demethylation, which is accelerated under alkaline conditions,^17^ the methoxy group is readily replaced by a hydroxyl group. For scopoletin, this oxidation reaction^39^ can lead to a partial transformation to esculetin, as supported by electrochemistry-mass spectrometry (EC-MS) measurements.^17^

### Interactions between Iron and Coumarins

Although our results suggest that the stability and redox reactivity of Fe–coumarin complexes strongly depend on the solution pH and the type of coumarin, the thermodynamic data to support this are not yet available. Further investigations into the role of coumarins in Fe acquisition strategies would greatly benefit from equilibrium constants for the formation of metal coumarin complexes, especially for Fe(II) and Fe(III).

Complexation of Fe protected fraxetin against oxidative degradation. As shown in Figure S28, Fe(III) complexation leads to a shift in oxidation potential of fraxetin to a higher potential, at both pH 5 and 8.5, implying that it becomes harder to oxidize the coumarin once it is complexed to Fe. In turn, formation of stable Fe–coumarin complexes prevented Fe from precipitating as Fe(III) hydroxide minerals. The complexation of Fe by catechol-bearing ligands and the redox stability of the resulting complexes have been widely studied.^52−54^

Catecholate ligands like fraxetin and esculetin are known to form stable complexes with Fe(III).^55^ Previously, it was suggested that the catechol moiety in coumarins may also form transient complexes with Fe(II), but only as a precursor reaction for the oxidation of Fe(II) to Fe(III) in order to form a stable Fe(III) complex, even under anoxic conditions.^14^ The results from our spectroscopic analysis and Ferrozine assays, however, demonstrate that esculetin and fraxetin can form Fe(II) complexes, at pH 7 and 8.5 and to a lesser degree at pH 6, and that this Fe(II) could be quantitatively recovered with Ferrozine. For the results demonstrated in Figures 6 and S21, the time between preparation of Fe(II)–coumarins complexes and Ferrozine addition was relatively short (ca. 1 min). However, when Ferrozine was added 1 day after the preparation of Fe(II)/Fe(III)–esculetin complex solutions that had been kept under anaerobic conditions, the recovered Fe(II) concentrations for the freshly prepared and the 1 day old solutions differed only by up to 5% (Figure S27). Also, scopoletin formed stable Fe complexes, in agreement with earlier observations,^8,17^ despite the fact that it does not have a catechol moiety. 1:2 and 1:3 Fe(II)–scopoletin complexes were identified after interaction of scopoletin with Fe(III)(hydr)oxide minerals.^17^ Also, partial oxidative demethylation of scopoletin to esculetin was observed in this study. It is unclear if this process occurred in our purely aqueous systems, yet if it did, it was fast and only to a limited extent: at pH 7 and 8.5, the spectra for Fe–scopoletin did not change over time but did distinctly differ from the Fe–esculetin spectra (Figures S16–S17 and S19–S20). How exactly scopoletin is coordinated to Fe is still unclear.

Interestingly, at pH 7 and 8.5 for all three coumarins, the spectra for Fe(II) and Fe(III) complexes were nearly identical (Figures 4, S13, and 14), suggesting a very similar coordination environment and redox state of the complexed Fe; Ferrozine was only reactive toward the Fe(II)–coumarin complexes. This implies that no net reduction of Fe(III) had occurred as a result of complexation and that the net redox state of the applied Fe had been preserved. As catecholate ligands tend to form very stable complexes with Fe(III),^44^ possibly, an electron from Fe(II) was delocalized over the unsaturated rings of the coumarins participating in the coordination complex, creating a similar coordination environment for the Fe(II) and Fe(III) complexes.

### Effect of Ferrozine on the Fe Redox State in Solution Containing Fe–Coumarin Complexes

When applied to Fe–coumarin solutions, Ferrozine can, depending on pH and coumarin, strongly affect the redox state of the Fe. Particularly, below neutral pH, Ferrozine induced Fe(III) reduction. Our MS analyses of Fe(III)–esculetin solutions indicate 1:3 complexes at pH 8.5 but 1:2 complexes at pH 6.5 (Figure S1). For 1:2 complexes, only four of six positions in the primary coordination sphere of Fe are occupied by esculetin, potentially making complexed Fe(III) more susceptible to reduction. Possibly, Ferrozine addition leads to the formation of Ferrozine–Fe(III)–coumarin complexes, facilitating Fe(III) reduction to Fe(II) and displacement of complexed coumarins through ligand exchange. Furthermore, Fe(III) reduction rates were smaller for Fe(III)–esculetin and Fe(III)–scopoletin than for Fe(III)–fraxetin, which has a lower redox potential.

## Conclusions

Because of the high redox reactivity of coumarins, it is critical to avoid experimental and analytical artifacts, while striving to elucidate the mechanisms by which exuded coumarins support plant growth, e.g., by enhancing the bioavailability of Fe in the rhizosphere. In this study, we particularly focused on coumarin degradation and the assessment of the redox state of Fe in solutions containing coumarins.

To prevent degradation while preparing coumarin (stock) solution by increasing the pH, care should be taken not to increase the pH further than necessary. The pH value at which degradation starts to occur depends on the coumarin: under oxic conditions, fraxetin proved susceptible to degradation already at lower pH values (>7) than scopoletin and esculetin (>10.5). The degradation rate increased more than proportionally with the coumarin concentration, implying an overall reaction order larger than one. Degradation under oxic conditions, e.g., as a result of a temporary excessive increase in pH, proved irreversible. It is strongly preferable to prepare coumarin solutions under anoxic conditions, especially for fraxetin, as degradation progresses much more slowly in absence of oxygen; for low coumarin concentrations (42 μM), degradation at pH 10.5 was limited, yet at pH 12.5, at least 40–75% of the coumarin degraded within 24 h, even under anoxic conditions. Based on how the spectra evolved over time under oxic and anoxic conditions, the degradation pathways appear to be different. The complexation of metals can protect coumarin ligands from degradation under oxic conditions in the soil pH range, as was illustrated for fraxetin complexed to Fe. In turn, coumarins formed soluble complexes with both Fe(II) and Fe(III), preserving the net redox state of the Fe and keeping it in solution under oxic conditions at circumneutral pH, where it would otherwise precipitate as Fe(III) hydroxide minerals.

The Ferrozine assay can be used to establish the redox speciation of Fe mobilized by coumarins but not for all coumarins under all conditions, and spectral interferences need to be accounted for. For the circumneutral pH range of 6–8.5, where soil Fe availability is limited and coumarin exudation is upregulated, Fe(II) in coumarin solutions could be quantitatively recovered. Fe(III)–coumarin complexes have absorbance at 563 nm, the wavelength used for establishing the Fe(II) concentration in the Ferrozine assay. Therefore, this interference needs to be corrected for the correct determination of the Fe(II) concentration. Ferrozine can affect the redox speciation of Fe in solutions with coumarins, e.g., by facilitating reduction of complexed Fe(III). Reduction rates increased with decreasing pH and were larger for fraxetin than for scopoletin and esculetin. Hence, to accurately determine Fe redox speciation, it is essential to measure absorbance within minutes after Ferrozine addition; for fraxetin at pH 6, reduction was so fast that determining the redox speciation through the Ferrozine assay proved not to be feasible. Under anoxic conditions, Fe redox speciation hardly changed over 24 h. Until it is verified if this also applies for oxic conditions, it is advisable to carry out the Ferrozine assay directly after sampling. For environmental samples containing Fe–coumarin complexes, the influence of other (redox-active) solutes like DOC on the accuracy of the Ferrozine assay needs to be further explored. Yet, for model systems, our study validates the Ferrozine assay as a valuable tool for unraveling the Fe dissolution mechanisms involved with coumarin-mediated Fe acquisition.

## Supporting Information Available

LC-MS measurements of Fe–coumarin complexes, UV–vis absorbances of coumarins at different pH, the molar extinction coefficients of coumarins, changes in UV–vis absorbance of coumarin under oxic and anoxic conditions at different pH, comparisons of UV–vis absorbances between coumarin in water and methanol, UV–vis absorbances of Fe–coumarin complexes under oxic and anoxic conditions at different pH, the changes in Fe speciation of Fe(II)–coumarin and Fe(III)–coumarin complexes in the presence and absence of Ferrozine, and the procedure for calculating the initial Fe(III) reduction rates and electrochemistry of coumarins and Fe(III)–coumarin complexes (PDF)

## References

[ref1] Borges F.; Roleira F.; Milhazes N.; Santana L.; Uriarte E. Simple coumarins and analogues in medicinal chemistry: Occurrence, synthesis and biological activity. Current Medicinal Chemistry. 2005, 12 (8), 887–916. 10.2174/0929867053507315. 15853704

[ref2] Stringlis I. A.; de Jonge R.; Pieterse C. M. J. The Age of Coumarins in Plant-Microbe Interactions. Plant Cell Physiol. 2019, 60 (7), 1405–1419. 10.1093/pcp/pcz076. 31076771 PMC6915228

[ref3] Rajniak J.; Giehl R. F. H.; Chang E.; Murgia I.; von Wiren N.; Sattely E. S. Biosynthesis of redox-active metabolites in response to iron deficiency in plants. Nature Chemical Biology. 2018, 14 (5), 442–450. 10.1038/s41589-018-0019-2. 29581584 PMC6693505

[ref4] Tsai H. H.; Rodriguez-Celma J.; Lan P.; Wu Y. C.; Velez-Bermudez I. C.; Schmidt W. Scopoletin 8-Hydroxylase-Mediated Fraxetin Production Is Crucial for Iron Mobilization. Plant Physiology. 2018, 177 (1), 194–207. 10.1104/pp.18.00178. 29559590 PMC5933141

[ref5] Tsai H. H.; Schmidt W. Mobilization of Iron by Plant-Borne Coumarins. Trends in Plant Science. 2017, 22 (6), 538–548. 10.1016/j.tplants.2017.03.008. 28385337

[ref6] Voges M.; Bai Y.; Schulze-Lefert P.; Sattely E. S. Plant-derived coumarins shape the composition of an Arabidopsis synthetic root microbiome. Proceedings of the National Academy of Sciences of the United States of America. 2019, 116 (25), 12558–12565. 10.1073/pnas.1820691116. 31152139 PMC6589675

[ref7] Siso-Terraza P.; Luis-Villarroya A.; Fourcroy P.; et al. Accumulation and Secretion of Coumarinolignans and other Coumarins in Arabidopsis thaliana Roots in Response to Iron Deficiency at High pH. Frontiers in Plant Science. 2016, 7, 22 10.3389/fpls.2016.01711. 27933069 PMC5120119

[ref8] Schmidt H.; Gunther C.; Weber M.; et al. Metabolome Analysis of Arabidopsis thaliana Roots Identifies a Key Metabolic Pathway for Iron Acquisition. Plos One. 2014, 9 (7), e102444 10.1371/journal.pone.0102444. 25058345 PMC4109925

[ref9] Rosenkranz T.; Oburger E.; Baune M.; Weber G.; Puschenreiter M. Root exudation of coumarins from soil-grown Arabidopsis thaliana in response to iron deficiency. Rhizosphere. 2021, 17, 100296 10.1016/j.rhisph.2020.100296.

[ref10] Siwinska J.; Siatkowska K.; Olry A.; et al. Scopoletin 8-hydroxylase: a novel enzyme involved in coumarin biosynthesis and iron-deficiency responses in Arabidopsis. Journal of Experimental Botany. 2018, 69 (7), 1735–1748. 10.1093/jxb/ery005. 29361149 PMC5888981

[ref11] Schmid N. B.; Giehl R. F. H.; Doll S.; et al. Feruloyl-CoA 6 ’-Hydroxylase1-Dependent Coumarins Mediate Iron Acquisition from Alkaline Substrates in Arabidopsis. Plant Physiology. 2014, 164 (1), 160–172. 10.1104/pp.113.228544. 24246380 PMC3875798

[ref12] Chutia R.; Abel S.; Ziegler J. Iron and Phosphate Deficiency Regulators Concertedly Control Coumarin Profiles in Arabidopsis thaliana Roots During Iron, Phosphate, and Combined Deficiencies. Frontiers in Plant Science. 2019, 10, 1 10.3389/fpls.2019.00113. 30804973 PMC6378295

[ref13] Kraemer S. M.; Crowley D. E.; Kretzschmar R. Geochemical aspects of phytosiderophore-promoted iron acquisition by plants. In Sparks D. L., Ed.; Advances in Agronomy; Elsevier Academic Press Inc.: San Diego, 2006; Vol 91, pp 1–46.

[ref14] Mladenka P.; Macakova K.; Zatloukalova L.; et al. In vitro interactions of coumarins with iron. Biochimie. 2010, 92 (9), 1108–1114. 10.1016/j.biochi.2010.03.025. 20381579

[ref15] Teres J.; Busoms S.; Martin L. P.; et al. Soil carbonate drives local adaptation in Arabidopsis thaliana. Plant Cell and Environment. 2019, 42 (8), 2384–2398. 10.1111/pce.13567. PMC6663613 31018012

[ref16] Robe K.; Conejero G.; Gao F.; et al. Coumarin accumulation and trafficking in Arabidopsis thaliana: a complex and dynamic process. New Phytologist. 2021, 229 (4), 2062–2079. 10.1111/nph.17090. 33205512

[ref17] Baune M.; Kang K.; Schenkeveld W. D. C.; Kraemer S. M.; Hayen H.; Weber G. Importance of oxidation products in coumarin-mediated Fe(hydr)oxide mineral dissolution. Biometals. 2020, 33, 305 10.1007/s10534-020-00248-y. 33015746

[ref18] Ziegler J.; Schmidt S.; Chutia R.; et al. Non-targeted profiling of semi-polar metabolites in Arabidopsis root exudates uncovers a role for coumarin secretion and lignification during the local response to phosphate limitation. Journal of Experimental Botany. 2016, 67 (5), 1421–1432. 10.1093/jxb/erv539. 26685189 PMC4762384

[ref19] Hirano A.; Arakawa T.; Shiraki K. Arginine increases the solubility of coumarin: Comparison with salting-in and salting-out additives. Journal of Biochemistry. 2008, 144 (3), 363–369. 10.1093/jb/mvn078. 18583357

[ref20] Seshadri T. R.; Rao P. S. Geometrical inversion in the acids derived from the coumarins. Proc. Indian Acad. Sci. (Math. Sci.) 1936, 3, 293–296. 10.1007/BF03035669.

[ref21] Li A.; Andren A. W. Solubility of polzchlorinated-Biphenyle in water-Alcohol mistures. 1. experimental-data. Environ. Sci. Technol. 1994, 28 (1), 47–52. 10.1021/es00050a008. 22175832

[ref22] Hesleitner P.; Kallay N.; Matijevic E. Adsorption at solid liquid interfaces. 6. The effect of methanol and ethanol on the ionic equilibria at the hematite water interface. Langmuir. 1991, 7 (1), 178–184. 10.1021/la00049a032.

[ref23] Xue Y.; Traina S. J. Cosolvent effect on goethite surface protonation. Environmental Science & Technology. 1996, 30 (11), 3161–3166. 10.1021/es9507168.

[ref24] Streng W. H.; Hsi S. K.; Helms P. E.; Tan H. G. H. General treatment of pH-solubility profiles of weak acids and bases and the effects of different acids on the solubility of a weak base. J. Pharm. Sci. 1984, 73 (12), 1679–1684. 10.1002/jps.2600731203. 6527235

[ref25] Stookey L. L. Ferrozine - A New spectrophotometric reagent for iron. Anal. Chem. 1970, 42 (7), 779–781. 10.1021/ac60289a016.

[ref26] Jeitner T. M. Optimized ferrozine-based assay for dissolved iron. Anal. Biochem. 2014, 454, 36–37. 10.1016/j.ab.2014.02.026. 24632099

[ref27] Statham P. J.; Jacobson Y.; van den Berg C. M. G. The measurement of organically complexed Fe-II in natural waters using competitive ligand reverse titration. Anal. Chim. Acta 2012, 743, 111–116. 10.1016/j.aca.2012.07.014. 22882830

[ref28] Willey J. D.; Kieber R. J.; Seaton P. J.; Miller C. Rainwater as a source of Fe(II)-stabilizing ligands to seawater. Limnology and Oceanography. 2008, 53 (4), 1678–1684. 10.4319/lo.2008.53.4.1678.

[ref29] Viollier E.; Inglett P. W.; Hunter K.; Roychoudhury A. N.; Van Cappellen P. The ferrozine method revisited: Fe(II)/Fe(III) determination in natural waters. Appl. Geochem. 2000, 15 (6), 785–790. 10.1016/S0883-2927(99)00097-9.

[ref30] Ammari T.; Mengel K. Total soluble Fe in soil solutions of chemically different soils. Geoderma. 2006, 136 (3–4), 876–885. 10.1016/j.geoderma.2006.06.013.

[ref31] Clemens S.; Weber M. The essential role of coumarin secretion for Fe acquisition from alkaline soil. Plant Signaling Behavior 2016, 11 (2), e1114197 10.1080/15592324.2015.1114197. 26618918 PMC4883844

[ref32] Mao Y. P.; Zhang M. M.; Xu J. Limitation of ferrozine method for Fe(II) detection: reduction kinetics of micromolar concentration of Fe(III) by ferrozine in the dark. International Journal of Environmental Analytical Chemistry. 2015, 95 (15), 1424–1434. 10.1080/03067319.2015.1114107.

[ref33] Pham H. T.; Yoo J.; VandenBerg M.; Muyskens M. A. Fluorescence of Scopoletin Including its Photoacidity and Large Stokes Shift. Journal of Fluorescence. 2020, 30 (1), 71–80. 10.1007/s10895-019-02471-4. 31872306

[ref34] Yu Q. Y.; Kandegedara A.; Xu Y. P.; Rorabacher D. B. Avoiding interferences from Good’s buffers: A contiguous series of noncomplexing tertiary amine buffers covering the entire range of pH 3–11. Anal. Biochem. 1997, 253 (1), 50–56. 10.1006/abio.1997.2349. 9356141

[ref35] Ferreira C. M. H.; Pinto I. S. S.; Soares E. V.; Soares H. (Un)suitability of the use of pH buffers in biological, biochemical and environmental studies and their interaction with metal ions - a review. Rsc Advances. 2015, 5 (39), 30989–31003. 10.1039/C4RA15453C.

[ref36] Jomova K.; Hudecova L.; Lauro P.; et al. A Switch between Antioxidant and Prooxidant Properties of the Phenolic Compounds Myricetin, Morin, 3 ’,4 ’-Dihydroxyflavone, Taxifolin and 4-Hydroxy-Coumarin in the Presence of Copper(II) Ions: A Spectroscopic, Absorption Titration and DNA Damage Study. Molecules. 2019, 24 (23), 4335 10.3390/molecules24234335. 31783535 PMC6930463

[ref37] Le Person A.; Moncomble A.; Cornard J. P. The Complexation of Al-III, Pb-II, and Cu-II Metal Ions by Esculetin: A Spectroscopic and Theoretical Approach. J. Phys. Chem. A 2014, 118 (14), 2646–2655. 10.1021/jp412291z. 24654741

[ref38] Pillar-Little E. A.; Zhou R.; Guzman M. I. Heterogeneous Oxidation of Catechol. J. Phys. Chem. A 2015, 119 (41), 10349–10359. 10.1021/acs.jpca.5b07914. 26403273

[ref39] Miller R. W.; Sirois J. C.; Morita H. Reaction of coumarins with horseradish-peroxidase. Plant Physiology 1975, 55 (1), 35–41. 10.1104/pp.55.1.35. 16659024 PMC541546

[ref40] Ciche T. A.; Blackburn M.; Carney J. R.; Ensign J. C. Photobactin: a catechol siderophore produced by Photorhabdus luminescens, an entomopathogen mutually associated with Heterorhabditis bacteriophora NC1 nematodes. Appl. Environ. Microbiol. 2003, 69 (8), 4706–4713. 10.1128/AEM.69.8.4706-4713.2003. 12902261 PMC169088

[ref41] Shubha J. P.; Puttaswamy Kinetics and mechanism of oxidation of coumarin and substituted coumarin-B in hydrochloric acid medium. Progress in Reaction Kinetics and Mechanism. 2008, 33 (4), 313–330. 10.3184/146867808X372604.

[ref42] Andjelkovic M.; Van Camp J.; De Meulenaer B.; et al. Iron-chelation properties of phenolic acids bearing catechol and galloyl groups. Food Chem. 2006, 98 (1), 23–31. 10.1016/j.foodchem.2005.05.044.

[ref43] Jones S. E.; Leon L. E.; Sawyer D. T. Redox chemistry of metal-catechol complexes in aprotic media. 2. 3,5-Di-tert-butylcatecholato and orthosemiquinonato complexes of iron(III). Inorg. Chem. 1982, 21 (10), 3692–3698. 10.1021/ic00140a019.

[ref44] Kraemer S. M.; Duckworth O. W.; Harrington J. M.; Schenkeveld W. D. C. Metallophores and Trace Metal Biogeochemistry. Aquatic Geochemistry. 2015, 21 (2–4), 159–195. 10.1007/s10498-014-9246-7.

[ref45] Schenkeveld W. D. C.; Schindlegger Y.; Oburger E.; Puschenreiter M.; Hann S.; Kraemer S. M. Geochemical Processes Constraining Iron Uptake in Strategy II Fe Acquisition. Environmental Science & Technology. 2014, 48 (21), 12662–12670. 10.1021/es5031728. 25275965 PMC4224094

[ref46] Sorensen J.; Thorling L. Stimulation by lepidocrocite of Fe(II)-dependent nitrite reduction. Geochimica Et Cosmochimica Acta. 1991, 55 (5), 1289–1294. 10.1016/0016-7037(91)90307-Q.

[ref47] Boyer R. F.; Clark H. M.; Sanchez S. Solubilization of ferrihydrite iron by plant phenolics - a model for rhizosphere processes. Journal of Plant Nutrition 1989, 12 (5), 581–592. 10.1080/01904168909363975.

[ref48] Thuong P. T.; Hung T. M.; Ngoc T. M.; et al. Antioxidant Activities of Coumarins from Korean Medicinal Plants and their Structure-Activity Relationships. Phytotherapy Research. 2010, 24 (1), 101–106. 10.1002/ptr.2890. 19468986

[ref49] Song Y.; Buettner G. R. Thermodynamic and kinetic considerations for the reaction of semiquinone radicals to form superoxide and hydrogen peroxide. Free Radical Biol. Med. 2010, 49 (6), 919–962. 10.1016/j.freeradbiomed.2010.05.009. 20493944 PMC2936108

[ref50] Medina M. E.; Iuga C.; Alvarez-Idaboy J. R. Antioxidant activity of fraxetin and its regeneration in aqueous media. A density functional theory study. Rsc Advances 2014, 4 (95), 52920–52932. 10.1039/C4RA08394F.

[ref51] Chen J. X.; Yang J.; Ma L. L.; Li J.; Shahzad N.; Kim C. K. Structure-antioxidant activity relationship of methoxy, phenolic hydroxyl, and carboxylic acid groups of phenolic acids. Scientific Reports. 2020, 10 (1), 2611 10.1038/s41598-020-59451-z. 32054964 PMC7018807

[ref52] Yamahara R.; Ogo S.; Masuda H.; Watanabe Y. (Catecholato)iron(III) complexes: structural and functional models for the catechol-bound iron(III) form of catechol dioxygenases. Journal of Inorganic Biochemistry. 2002, 88 (3–4), 284–294. 10.1016/S0162-0134(01)00353-1. 11897342

[ref53] Mladenka P.; Kalinowski D. S.; Haskova P.; et al. The Novel Iron Chelator, 2-Pyridylcarboxaldehyde 2-Thiophenecarboxyl Hydrazone, Reduces Catecholamine-Mediated Myocardial Toxicity. Chem. Res. Toxicol. 2009, 22 (1), 208–217. 10.1021/tx800331j. 19172757

[ref54] Schweigert N.; Zehnder A. J. B.; Eggen R. I. L. Chemical properties of catechols and their molecular modes of toxic action in cells, from microorganisms to mammals. Environmental Microbiology. 2001, 3 (2), 81–91. 10.1046/j.1462-2920.2001.00176.x. 11321547

[ref55] Perron N. R.; Brumaghim J. L. A Review of the Antioxidant Mechanisms of Polyphenol Compounds Related to Iron Binding. Cell Biochemistry and Biophysics. 2009, 53 (2), 75–100. 10.1007/s12013-009-9043-x. 19184542

