# Supplementary material for: Stability of Coumarins and Determination of the Net Iron Oxidation State of Iron–Coumarin Complexes: Implications for Examining Plant Iron Acquisition Mechanisms
Source: ACS Earth Space Chem. 2023 Nov 10;7(12):2339–52. doi: 10.1021/acsearthspacechem.3c00199 (PMC10749481; doi:10.1021/acsearthspacechem.3c00199)

Stability of coumarins and determination of the net iron oxidation state of iron-coumarin complexes: implications for examining plant iron acquisition mechanisms

Kyounglim Kang[a†], Walter D.C. Schenkeveld[b*], Guenther Weber[c], Stephan M. Kraemer[a]

[a] Environmental Geochemistry, Centre for Microbiology and Environmental Systems Science, University of Vienna, 1090 Vienna, Austria

[b] Soil Chemistry and Chemical Soil Quality, Environmental Sciences, 6708 PB Wageningen University, Wageningen, The Netherlands

[c] Leibniz-Institut für Analytische Wissenschaften – ISAS, 44227 Dortmund, Germany

[†]current address: Department of Civil and Environmental Engineering, University of California, Davis, CA 95616, United States

Corresponding Author

* w.d.c.schenkeveld@wur.nl

Content

Figures	***contents***		***page***
**Figure. S1.**	Mass spectra of Fe(III)-esculetin complexes at pH 6.5 and 8.5	....	2
**Figure. S2.**	UV-Vis absorption spectra of fraxetin at different pH values	....	3
**Figure. S3.**	UV-Vis absorption spectra of scopoletin at different pH values	....	4
**Figure. S4.**	UV-Vis absorption spectra of esculetin at different pH values	....	5
**Figure. S5.**	Changes in UV-vis absorption spectra of three coumarin compounds at different concentrations at pH 12.5 under oxic conditions	....	7
**Figure. S6.**	Experimental setup of oxidation of three coumarin compounds	....	8
**Figure. S7.**	Effect of initial pH (12.5) on oxidation of three coumarin compounds under oxic conditions	....	9
**Figure. S8.**	Effects of initial pH (10.5) on oxidation of three coumarin compounds under oxic conditions	....	10
**Figure. S9.**	Changes in UV-vis absorption spectra of three coumarins at pH 10.5 under anoxic conditions	....	11
**Figure. S10.**	Effects of initial pH (12.5) on oxidation of three coumarin compounds under anoxic conditions	....	12
**Figure. S11.**	Comparison of UV–Vis absorption spectra of three coumarins dissolved in water and the spectra of the coumarins dissolved in 5 % methanol	....	13
**Figure. S12.**	The esculetin solution in the presence of Fe(II) or Fe(III) at pH 6, 7 and 8.5.	....	14
**Figure. S13.**	Changes in UV–Vis absorbance spectra of scopoletin in the presence and absence of Fe(II) or Fe(III) under anoxic conditions	....	15
**Figure. S14.**	Changes in UV–Vis absorbance spectra of esculetin in the presence and absence of Fe(II) or Fe(III) under anoxic conditions	....	16
**Figure. S15.**	Changes of UV–Vis absorption spectra of Fe(II)-fraxetin complexes under oxic conditions.	....	17
**Figure. S16.**	Changes of UV–Vis absorption spectra of Fe(II)-scopoletin complexes under oxic conditions.	....	17
**Figure. S17.**	Changes of UV–Vis absorption spectra of Fe(II)-esculetin complexes under oxic conditions.	....	18
**Figure. S18.**	Changes of UV–Vis absorption spectra of Fe(III)-fraxetin complexes under oxic and anoxic conditions.	....	19

**Figure. S19.**	Changes of UV–Vis absorption spectra of Fe(III)-scopoletin complexes under oxic and anoxic conditions.	....	20
**Figure. S20.**	Changes of UV–Vis absorption spectra of Fe(III)-esculetin complexes under oxic and anoxic conditions.	....	21
**Figure. S21.**	The UV–Vis absorption spectra of Ferrozine and Fe(III) + Ferrozine, Fe(II)+Ferrozine and Fe(II)+Fe(III)+Ferrozine at pH 6-8.5	....	22
**Figure. S22.**	Linear relations between the concentration of Fe(III)-coumarin complexes in the presence of a stoichiometric excess of coumarin	....	24
**Figure. S23.**	Change in UV–Vis absorbance spectra of Fe(III)-coumarin complexes in the presence of Ferrozine under anoxic condition.	....	26
**Figure. S24.**	Change in UV–Vis absorbance spectra of Fe(III)-Fe(II)-coumarin complexes in the presence of Ferrozine under anoxic condition.	....	28
**Figure. S25.**	Changes in Fe speciation of Fe(III)-Fe(II)-coumarin in the presence of Ferrozine under anoxic condition.	....	30
**Figure. S26.**	Procedure for calculating the initial Fe(III) reduction rates	....	31
**Figure. S27.**	Fe(II) recovery from Fe(III)-Fe(II)-esculetin by Ferrozine at pH 6, 7 and 8.5.	....	32
**Figure. S28.**	Electrochemistry of coumarins at pH 5	....	33

Tables

**Table. S1.**	Molar extinction coefficient and the local maximum wavelength of three coumarins	....	6
**Table. S2.**	Extents of degradation of three coumarins under oxic conditions	....	6
**Table. S3.**	Initial Fe(III) reduction rates	....	31

Figure S1. 50 µM Fe(III) + 200 µM esculetin in 25 mM ammonium bicarbonate adjusted to the desired pH (pH 6.5 and 8.5). Mass spectra were recorded in the *m/z* range 100-800 in positive electrospray mode using a Thermo Scientific LTQ-FT ultra instrument. The isotopic pattern of Fe in the complexes is in excellent agreement with the theoretically expected one, and the mass accuracy is better than 8 ppm.

(a) Fe(III)-esculetin complex (1:2) at pH 6.5

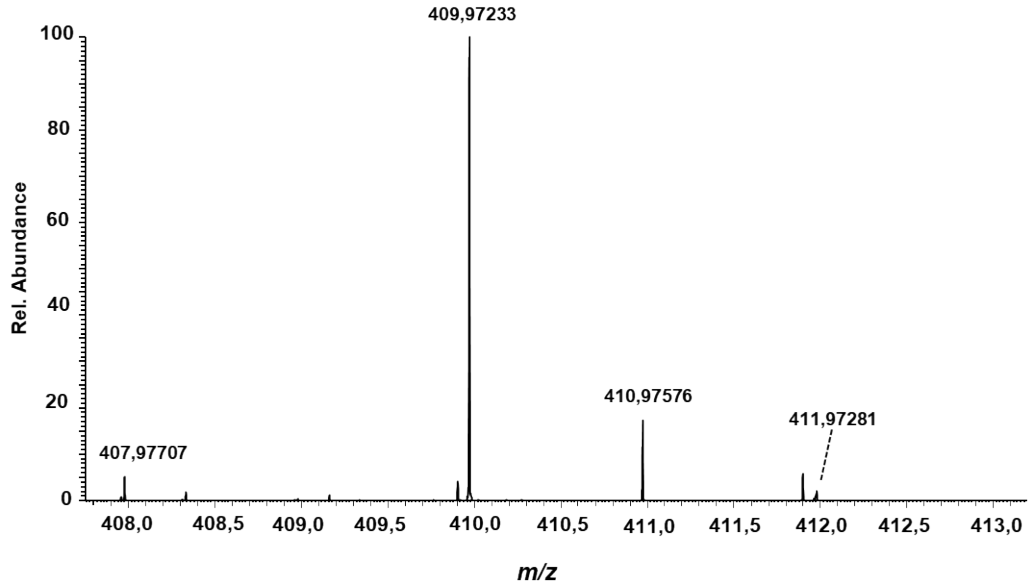

(b) Fe(III)-esculetin complex (1:3) at pH 8.5

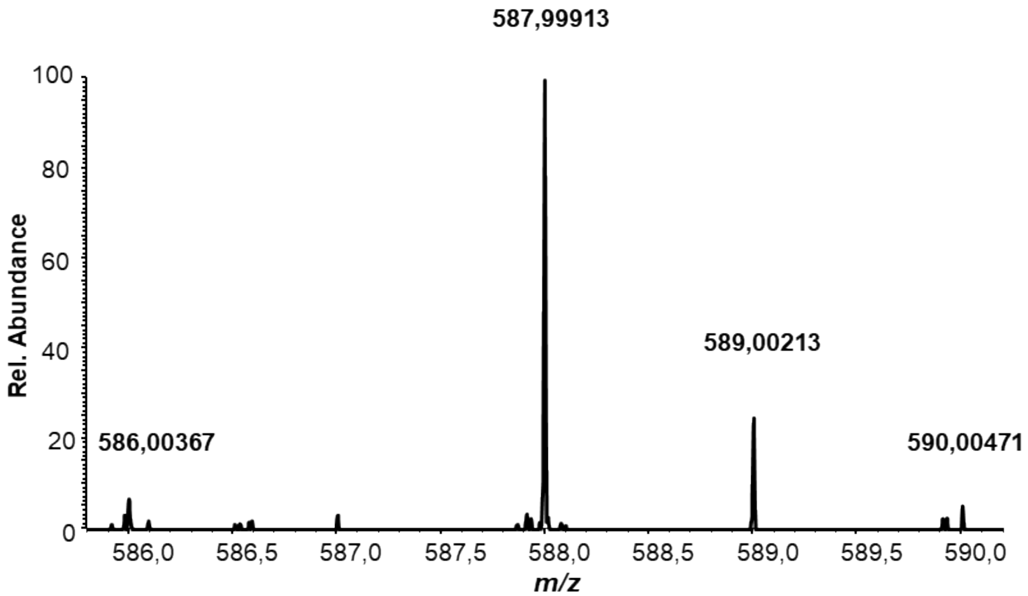

Figure S2. The UV–visible absorption (abs) spectra of fraxetin (frax) (initial concentration: 42 μM) at different pH values (a). The change of abs spectra of frax at pH (b) 11.5, (c) 10.5, (d) 9.5, (e) 8.5, (f) 7, (g) 6 and (h) 4 under oxic conditions over 24 h.

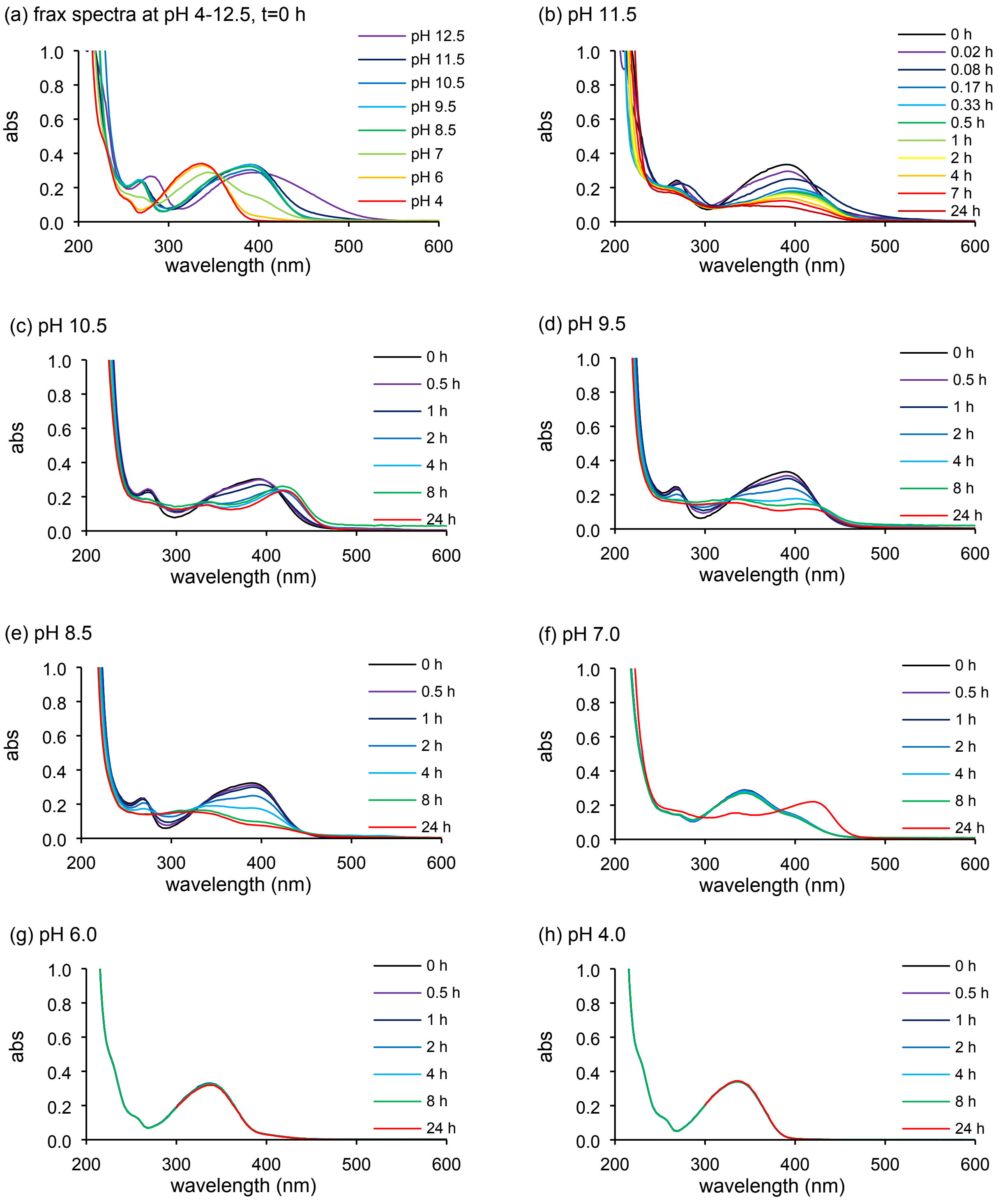

Figure S3. The UV–visible absorption (abs) spectra of scopoletin (scop) (initial concentration: 42 μM) at different pH values (a). The change of abs spectra of scop at pH (b) 11.5, (c) 10.5, (d) 9.5, (e) 8.5, (f) 7, (g) 6 and (h) 4 under oxic conditions over 24 h.

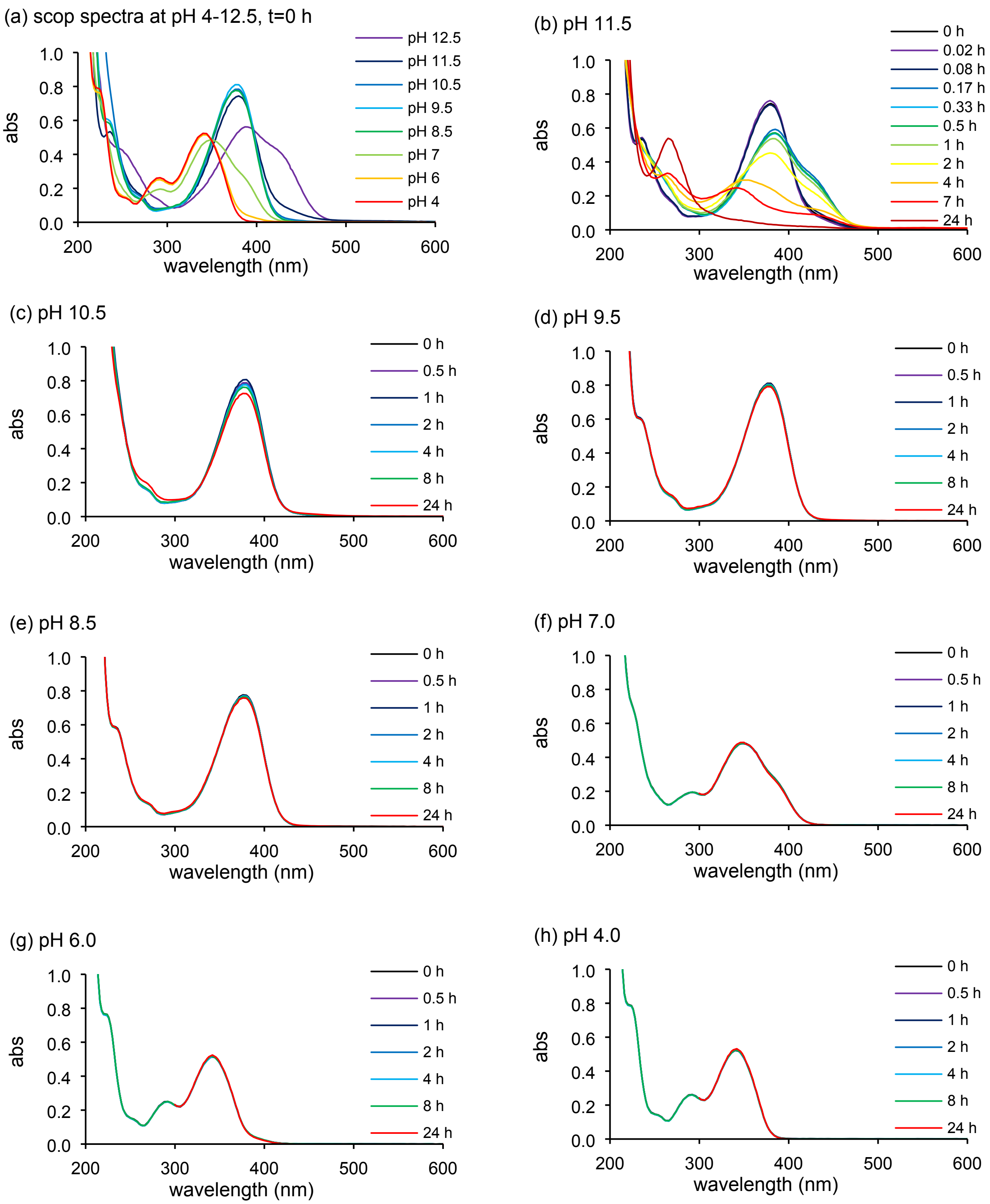

Figure S4. The UV–visible absorption (abs) spectra of esculetin (esc) (initial concentration: 42 μM) at different pH values (a). The change of abs spectra of esc at pH (b) 11.5, (c) 10.5, (d) 9.5, (e) 8.5, (f) 7, (g) 6 and (h) 4 under oxic conditions over 24 h.

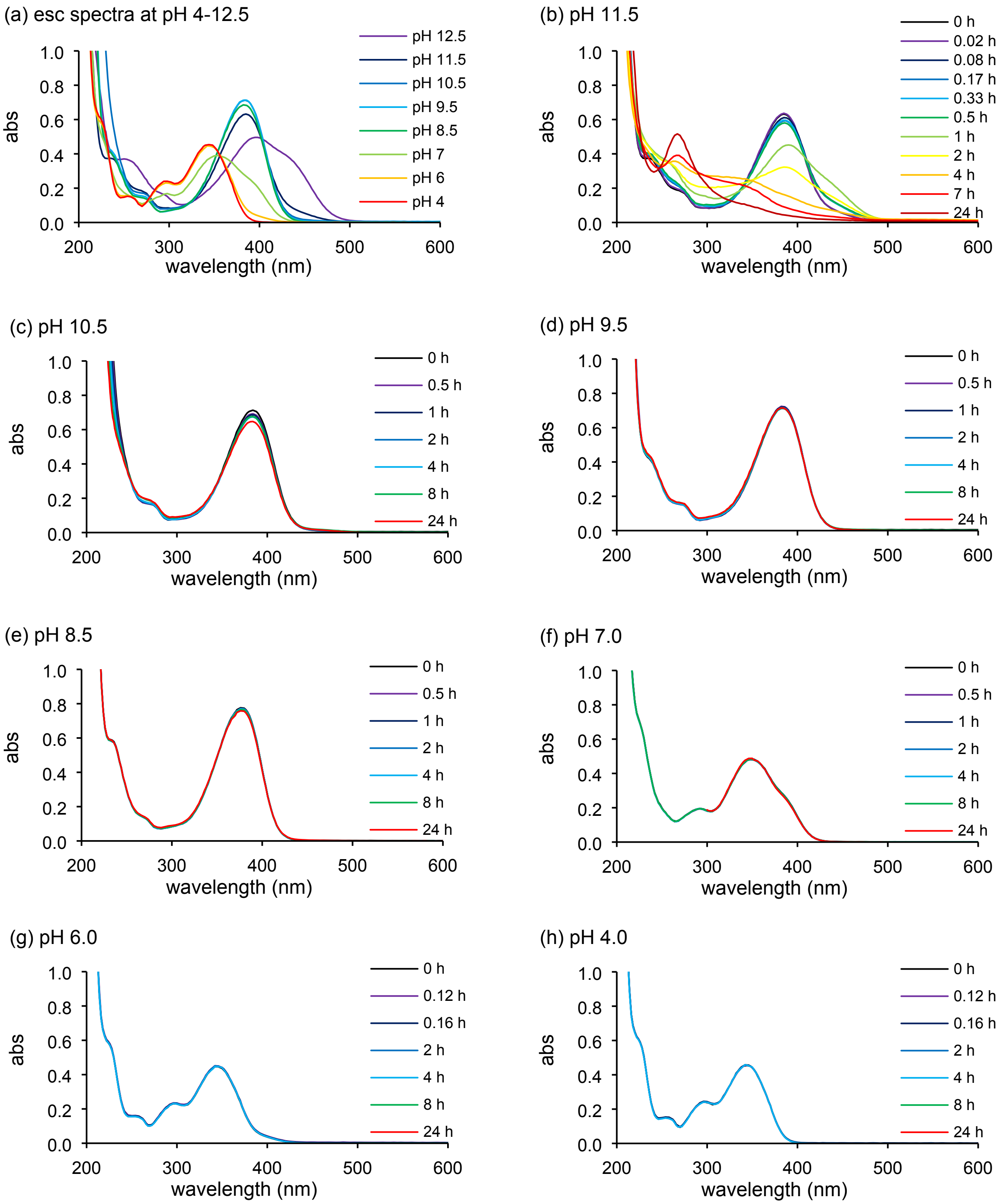

Table S1. The molar extinction coefficients (ε) and the local maximum wavelength (λ_{max}) as a function of pH for the three coumarins.

	frax		scop		esc	
	λ_{max} (nm)	ε (M^{-1} cm^{-1})	λ_{max} (nm)	ε (M^{-1} cm^{-1})	λ_{max} (nm)	ε (M^{-1} cm^{-1})
pH 12.5	393	6860	389	13400	396	11800
pH 11.5	390	7990	379	17700	385	15000
pH 10.5	390	7260	378	18700	384	17000
pH 9.5	390	7980	378	19400	384	17000
pH 8.5	390	7710	378	18600	382	16300
pH 7.0	344	6890	348	11500	352	9300
pH 6.0	338	7860	342	12300	344	10600
pH 4.0	338	8100	340	12500	342	10900

Table S2. The extent of degradation of coumarins ($Abs_{t=t}$/ $Abs_{t=0}$ * 100%) after 2 h under oxic conditions.

pH	frax (%)	scop (%)	esc (%)
12.5	46	23	47
11.5	53	40	49
10.5	31	0.54	5.3
9.5	29	0.82	0
8.5	23	0.55	0
7.0	0.97	0.58	0
6.0	0	0.402	0
4.0	0	0.98	0

Figure S5. Changes in UV–visible absorbance (abs) spectra of (a & b) frax, (c & d) scop and (e & f) esc (initial concentrations: 2.5 mM and 42 µM (black spectra)) over time at pH 12.5 under oxic conditions. For selected sampling times, samples were 60 times diluted (~ 42 µM).

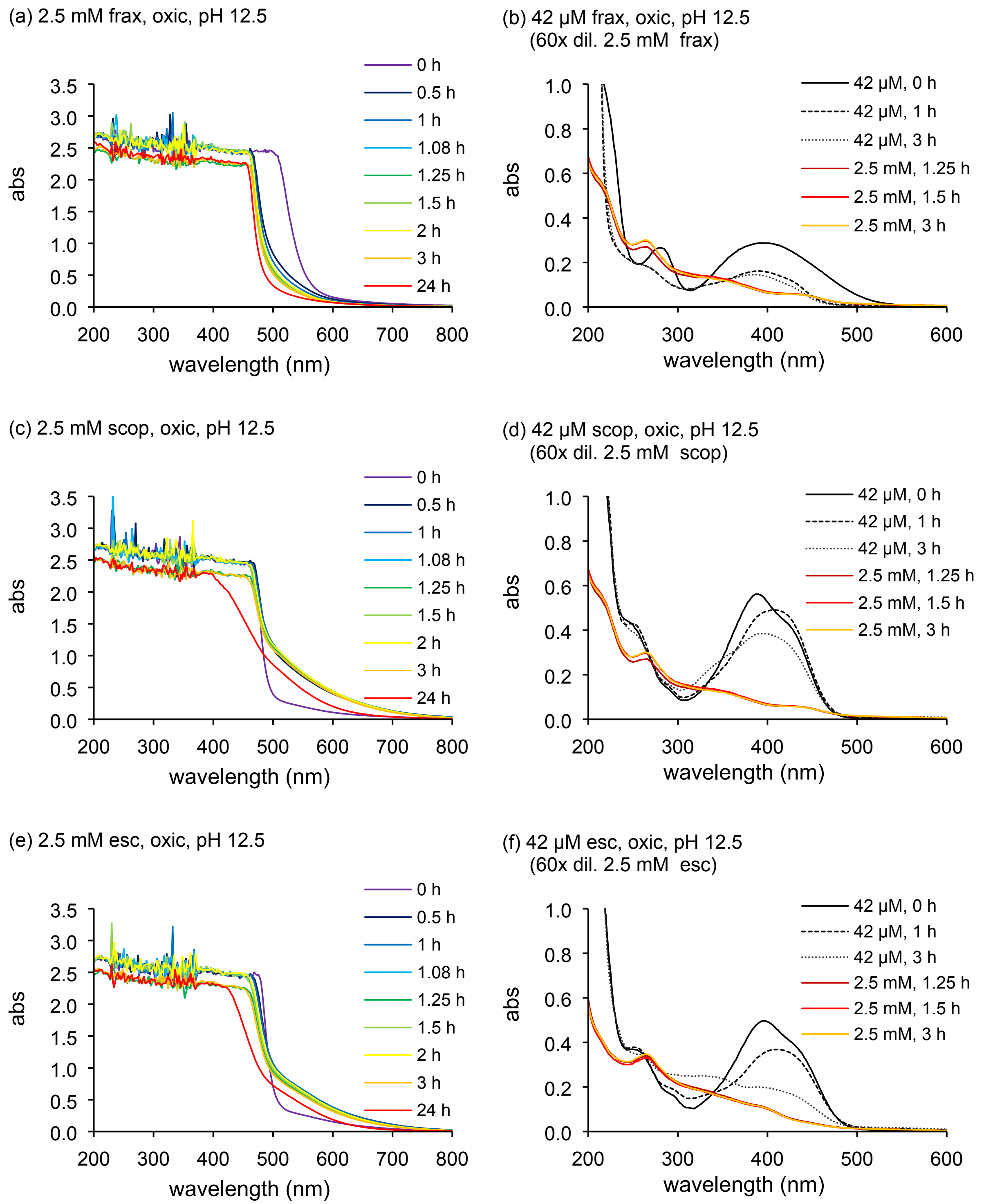

Figure S6. Setup of the experiments in which 2.5 mM solutions of esculetin (1,4), scopoletin (2,5) and fraxetin (3,6) are exposed to oxygen. In (a) all samples are at pH 12.5, in (b) the pH of sample 1-3 has been lowered to 8.5, whereas for 4-6 the pH had been maintained at 12.5.

(a) pH 12.5, t=0

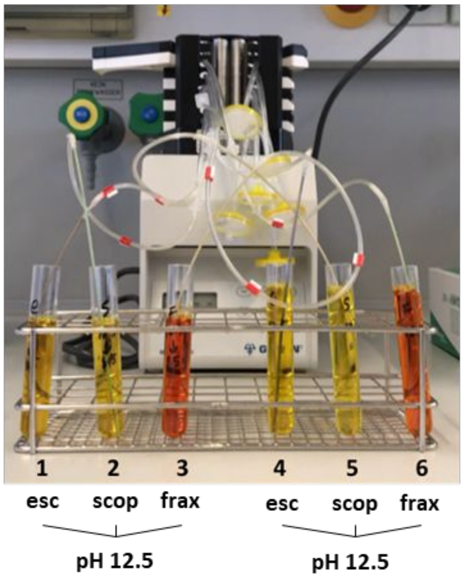

(b) pH 8.5 (1-3) and pH 12.5 (4-6), t=2 h

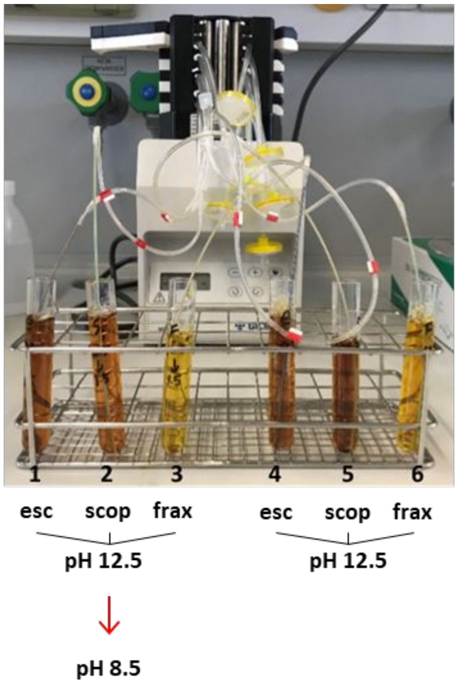

Figure S7. Changes in UV–visible absorbance (abs) spectra of frax and scop (initial concentration: 42 µM (a & c) and 2.5 mM (b & d)) over time at pH 8.5 under oxic conditions. For the coloured spectra, the solution pH had first been increased to 12.5 and had been maintained at this level for 2 hours before setting it to 8.5, all under oxic conditions; t = 0 corresponds to the moment the pH was set to 8.5 (first measurement time after 0.25 h). The results of control treatments (pH had not been increased) for pH 8.5 are presented as black spectra (reference spectra). For the 2.5 mM treatment (b & d) samples were diluted 60 times in order to examine the absorbance (< 1) between 200 to 600 nm.

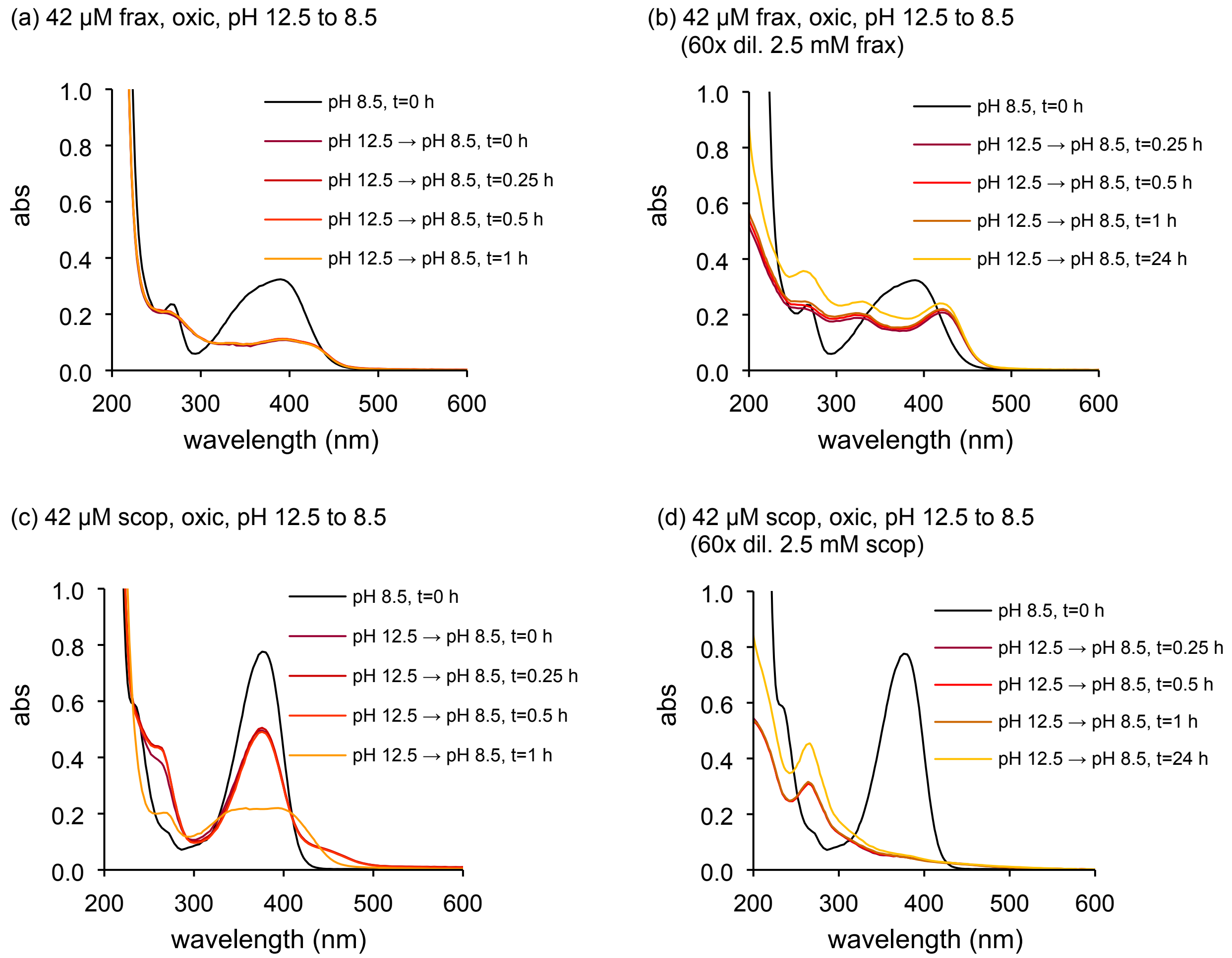

Figure S8. Changes in UV–visible absorbance (abs) spectra of (a) frax and (b) scop and (c) esc (initial concentration: 42 µM) over time at pH 8.5 under oxic conditions. For the coloured spectra, the solution pH had first been increased to 10.5 and had been maintained at this level for 2 hours before setting it to 8.5, all under oxic conditions; t = 0 corresponds to the moment the pH was set to 8.5 (first measurement time after 0.25 h). The results of control treatments (pH had not been increased) for pH 8.5 are presented as black spectra.

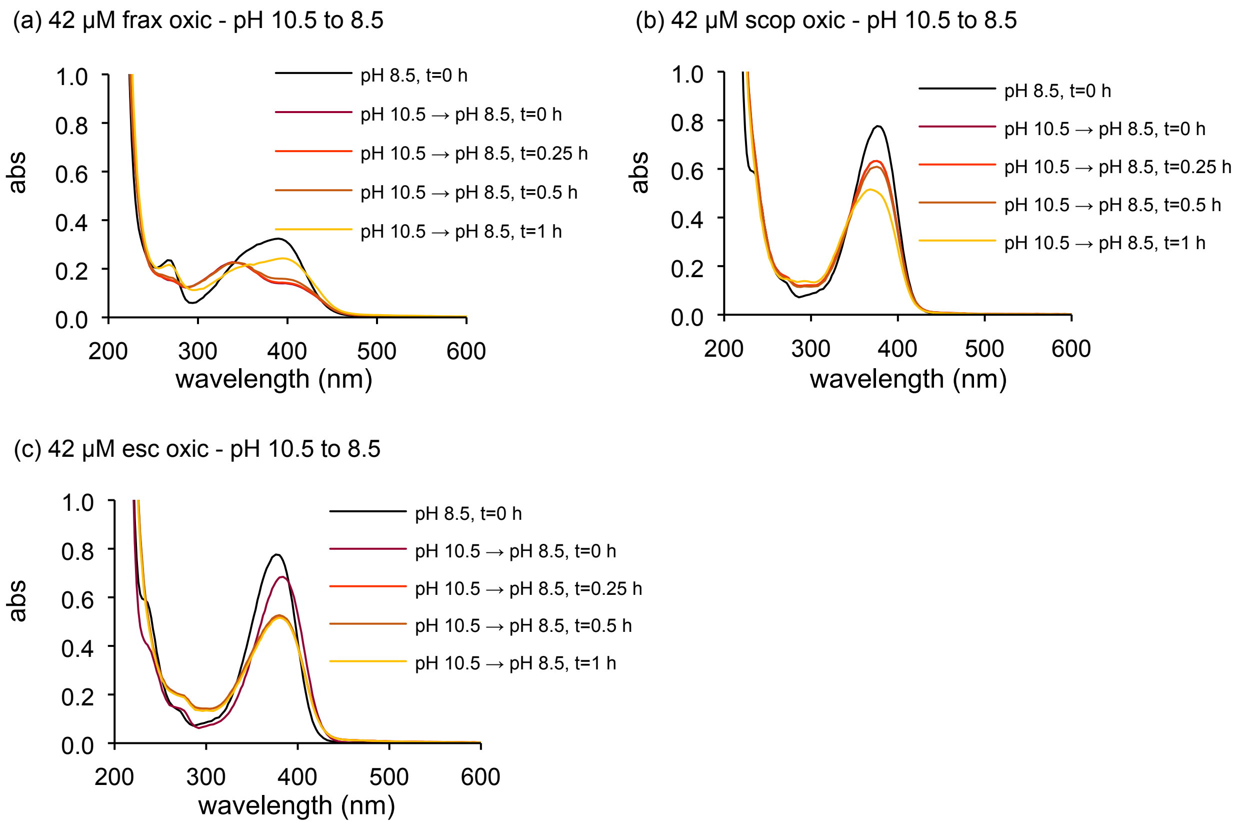

Figure S9. The change in UV–visible absorbance (abs) spectra of (a) frax, (b) scop and (c) esc (initial concentration: 42 μM) over time at pH 10.5 under anoxic conditions.

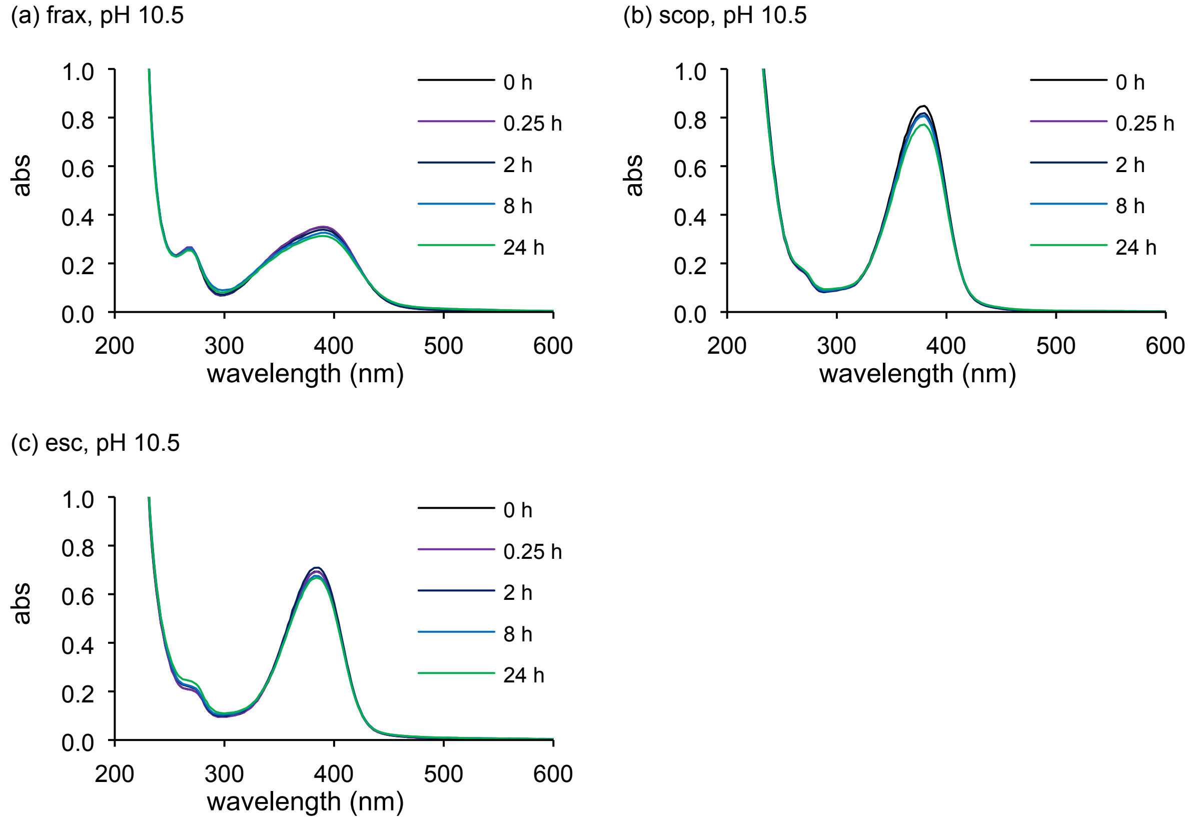

Figure S10. Changes in UV–visible absorbance (abs) spectra of (a & b) frax, (c & d) scop and (e & f) esc (initial concentration: 2.5 mM) at pH 8.5 under anoxic conditions. The solution pH had first been increased to 12.5 and had been maintained at this level for 2 hours before setting it to 8.5, all under anoxic conditions; t = 0 corresponds to the moment the pH was set to 8.5. The samples in (b, d and f) were diluted 60 times in order to examine the absorbance (< 1) between 200 to 600 nm.

(a) 2.5 mM frax anoxic-pH 12.5 to 8.5

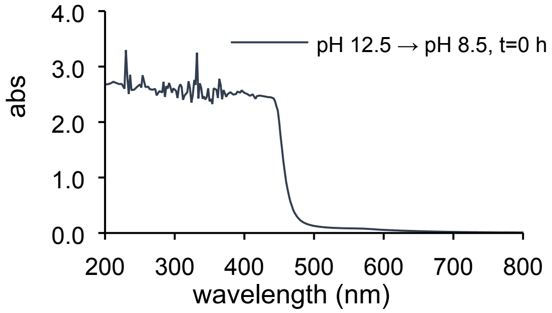

(b) 2.5 mM frax anoxic-pH 12.5 to 8.5
(60x dil. 2.5 mM frax)

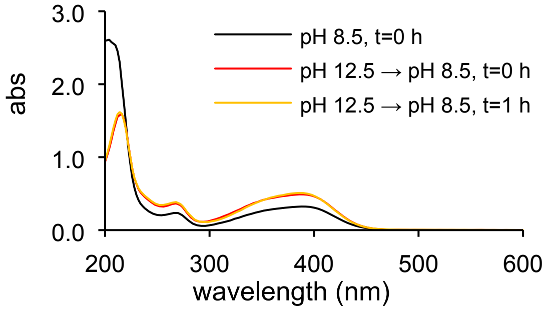

(c) 2.5 mM scop anoxic-pH 12.5 to 8.5

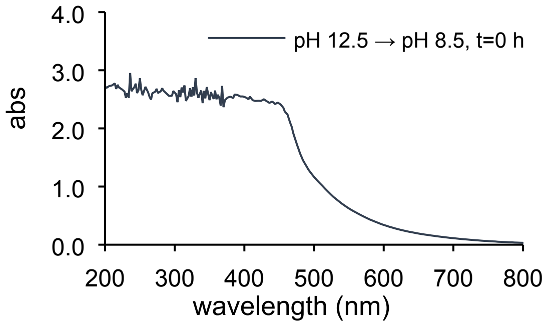

(d) 2.5 mM scop anoxic-pH 12.5 to 8.5
(60x dil. 2.5 mM scop)

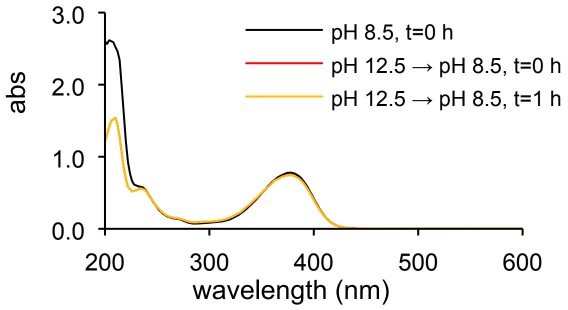

(e) 2.5 mM esc anoxic-pH 12.5 to 8.5

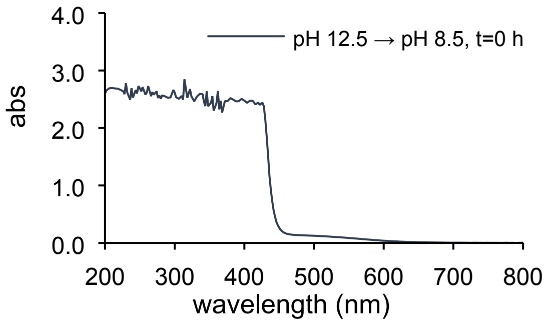

(f) 2.5 mM esc anoxic-pH 12.5 to 8.5
(60x dil. 2.5 mM frax)

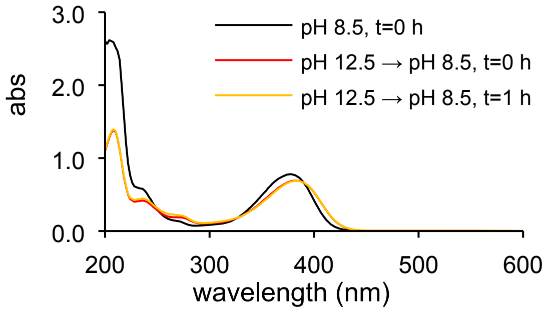

Figure S11. Comparison of UV–visible absorption (abs) spectra of the coumarins ((a) frax, (b) scop and (c) esc dissolved in water and the spectra of the coumarins dissolved in 5 % methanol (firstly coumarins were dissolved in pure methanol and water was added to a final methanol concentration of 5 %) under anoxic conditions at pH values. The abs of coumarins in 5 % methanol were analyzed 2 h after the mixing.

(a) frax

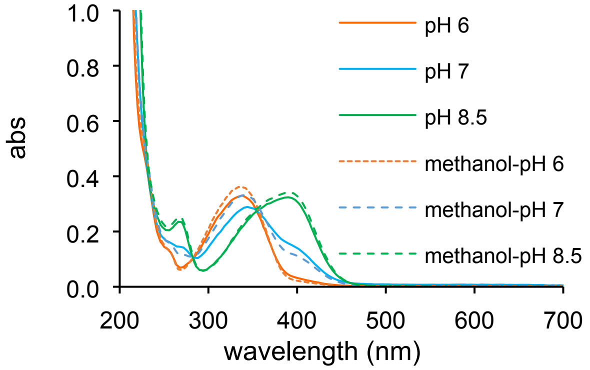

(b) scop

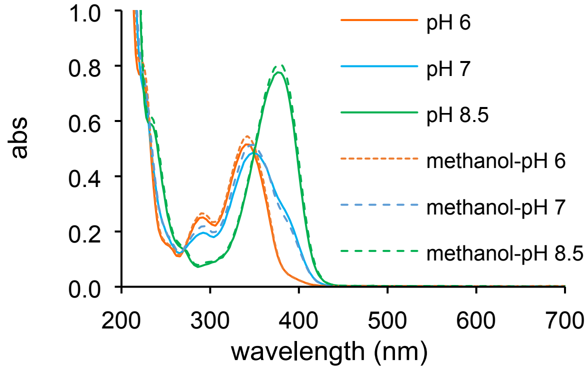

(c) esc

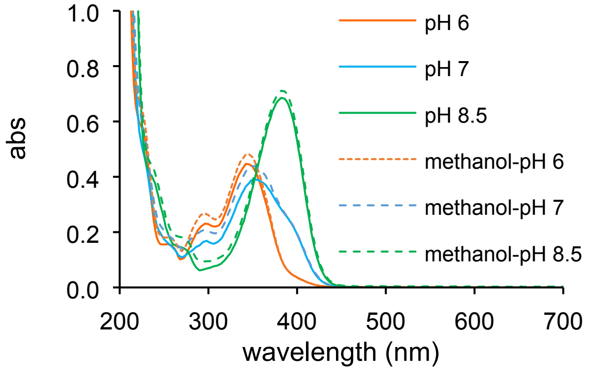

(d) 5 % methanol

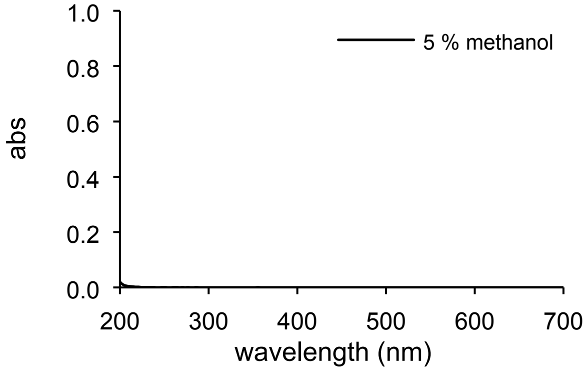

Figure S12. The esculetin solution (83 μM) in the presence of 10 μM Fe(II) or Fe(III) at pH 6, 7 and 8.5.

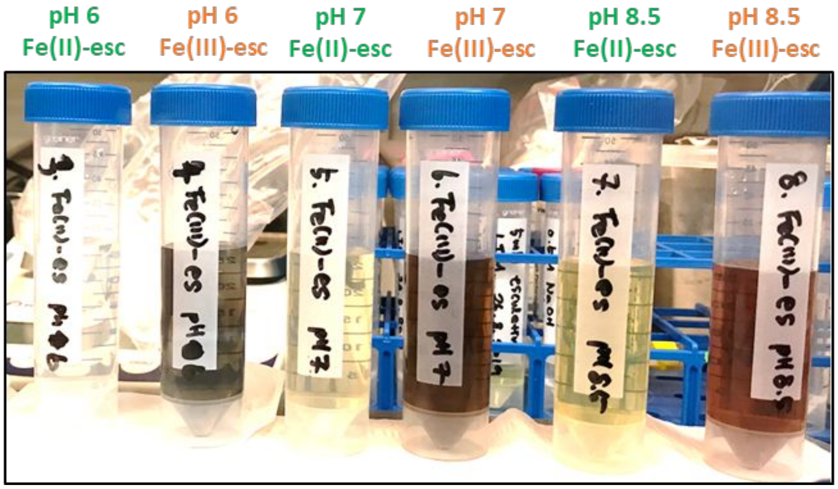

Figure S13. The change in UV–visible absorbance (abs) spectra of scopoletin (scop, initial concentration: 42 μM) in the presence and absence of 10 μM Fe(**II**) or Fe(**III**) at (a) pH 8.5, (b) pH 7.0 and (c) pH 6.0 under anoxic conditions (t=0 h: samples were analyzed immediately after taking the sample out of the glovebox).

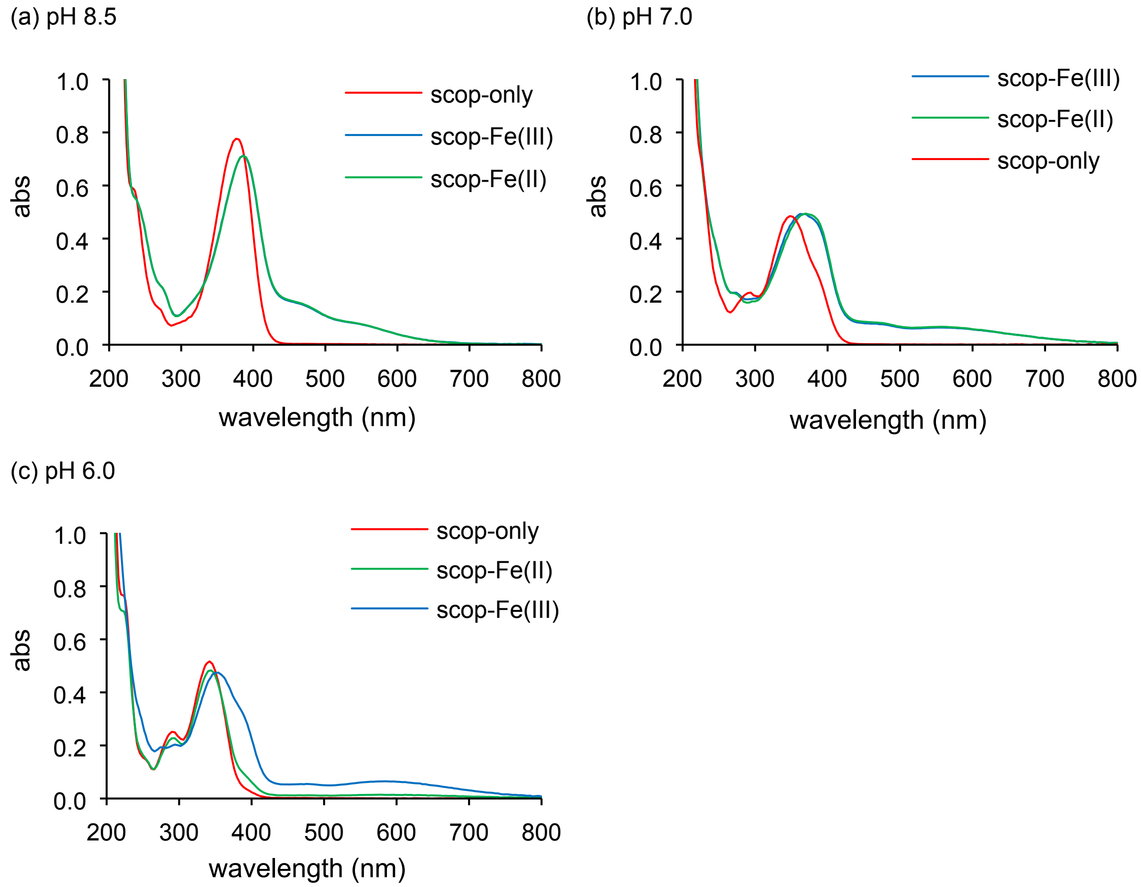

Figure S14. The change in UV–visible absorbance (abs) spectra of esculetin (esc, initial concentration: 42 μM) in the presence and absence of 10 μM Fe(**II**) or Fe(**III**) at (a) pH 8.5, (b) pH 7.0 and (c) pH 6.0 under anoxic conditions (t=0 h: samples were analyzed immediately after taking the sample out of the glovebox).

(a) pH 8.5

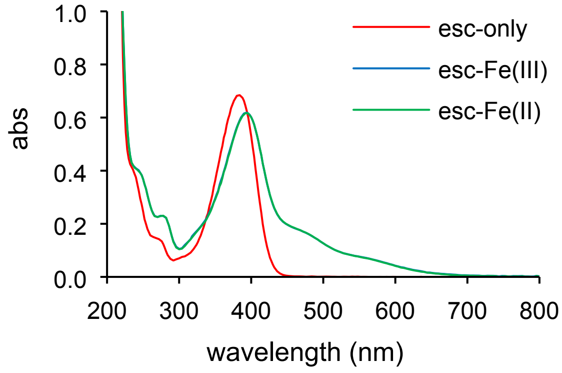

(b) pH 7.0

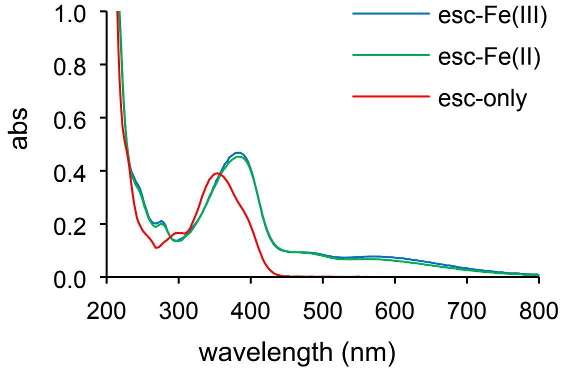

(b) pH 6.0

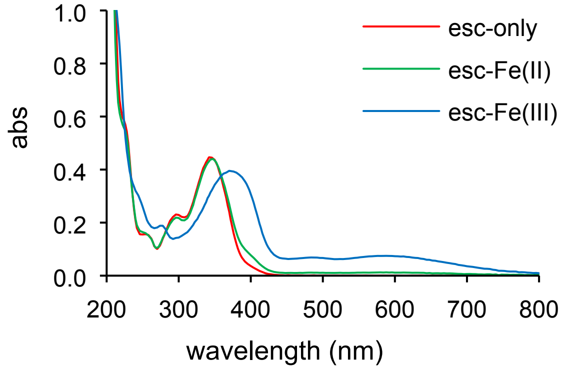

Figure S15. The change of UV–visible absorption (abs) spectra of Fe(II)-frax (10 μM Fe(II) and 42 μM frax) at pH (a) 8.5, (b) 7 and (c) 6 under oxic conditions.

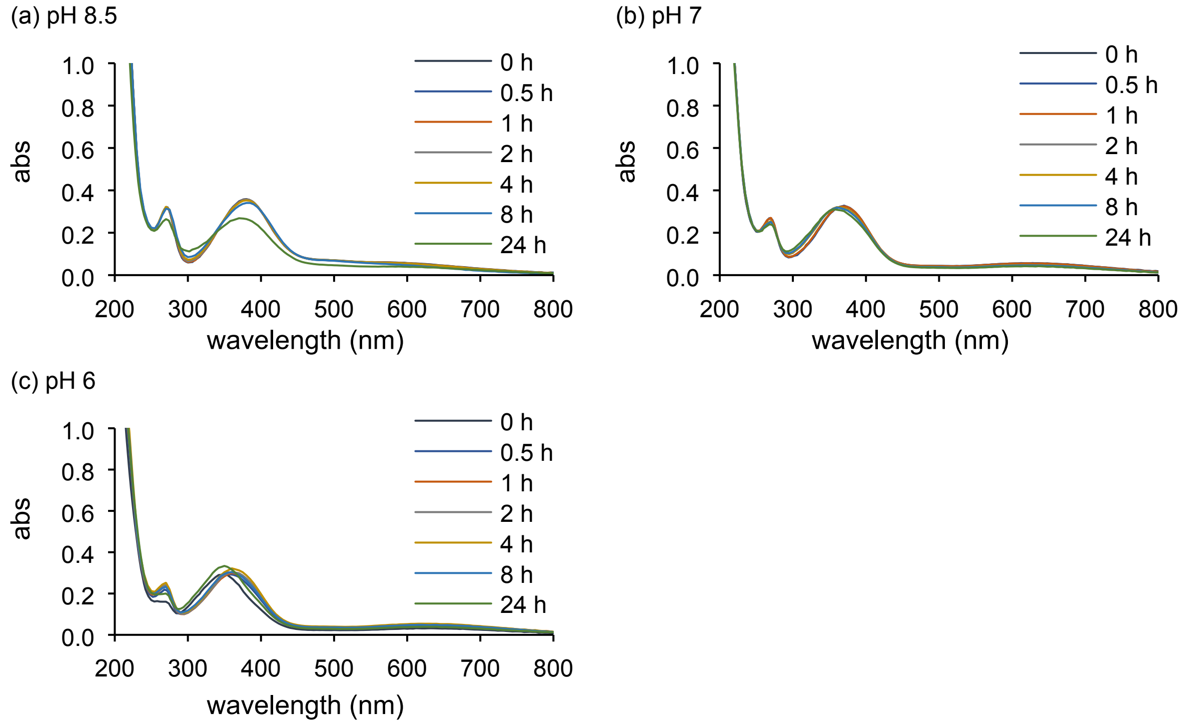

Figure S16. The change of UV–visible absorption (abs) spectra of Fe(II)-scop (10 μM Fe(II) and 42 μM scop) at pH (a) 8.5, (b) 7 and (c) 6 under oxic conditions.

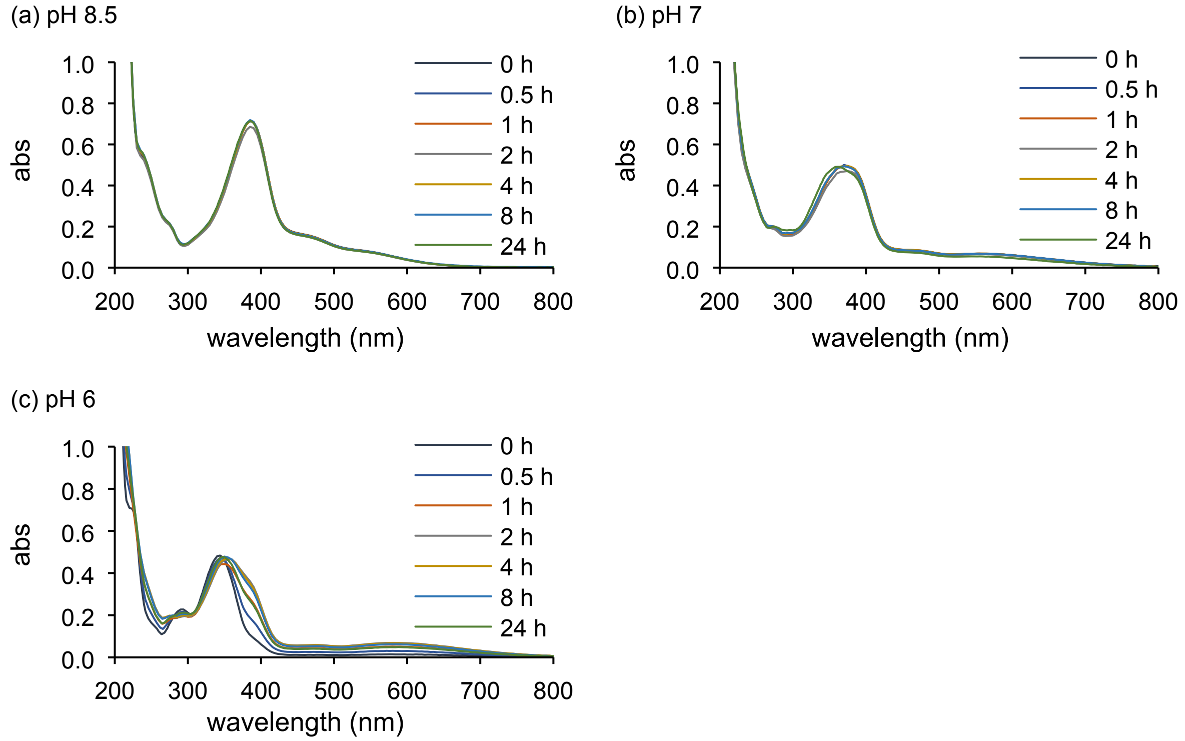

Figure S17. The change of UV–visible absorption (abs) spectra of Fe(II)-esc (10 μM Fe(II) and 42 μM esc) at pH (a) 8.5, (b) 7 and (c) 6 under oxic conditions.

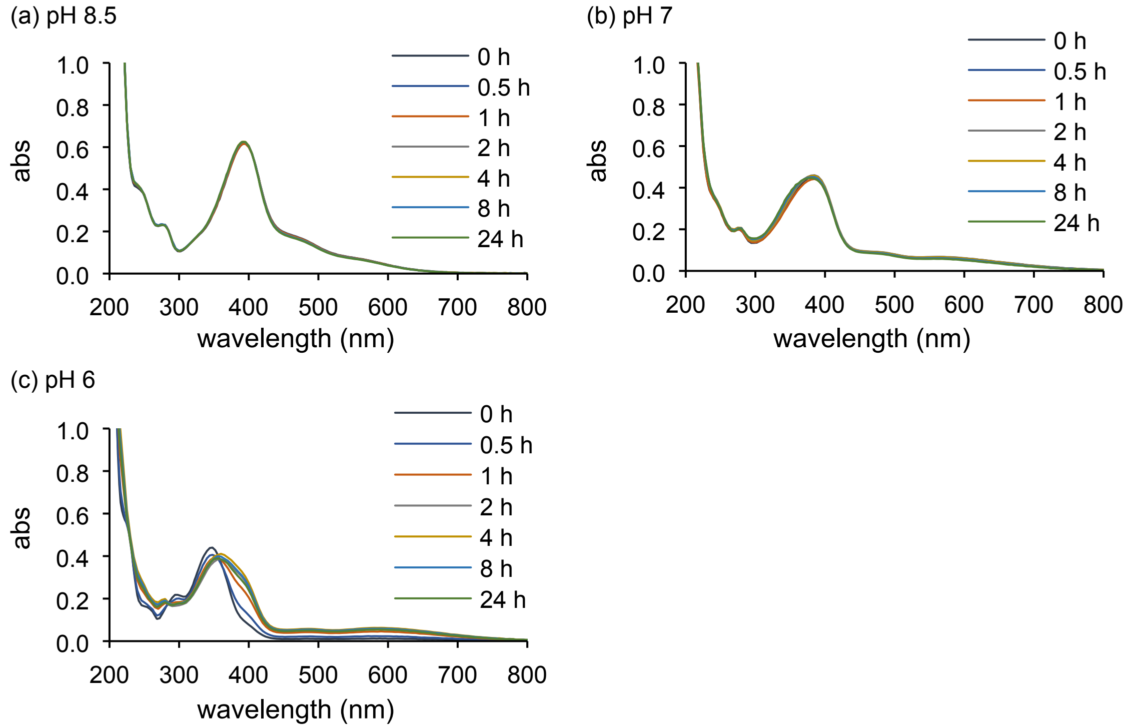

Figure S18. The change of UV–visible absorption (abs) spectra of Fe(III)-frax (10 μM Fe(III) and 42 μM frax) at pH (a) and (b) 8.5, (c) and (d) 7 and (e) and (f) 6 under anoxic and oxic conditions.

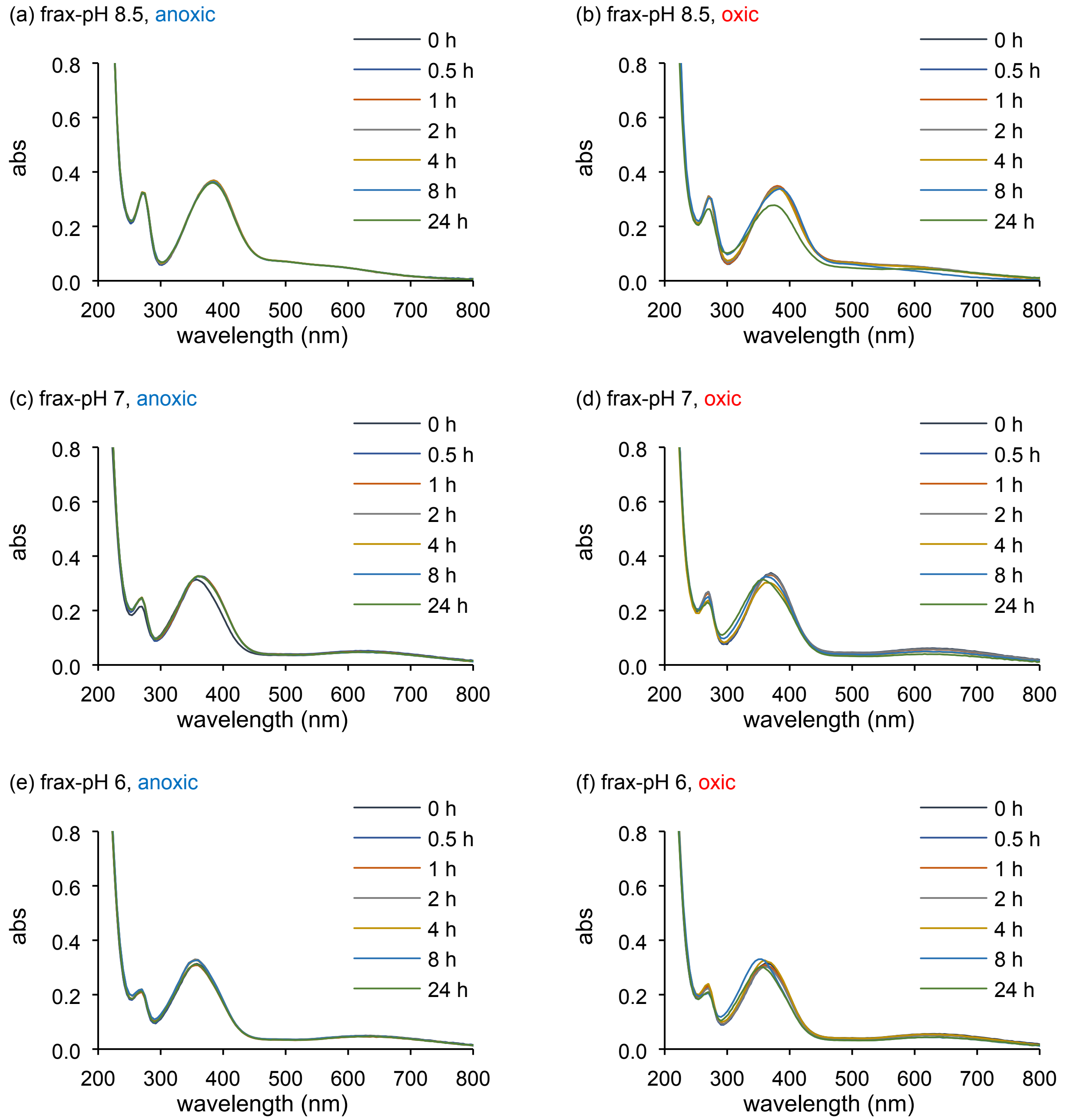

Figure S19. The change of UV–visible absorption (abs) spectra of Fe(III)-scop (10 μM Fe(III) and 42 μM scop) at pH (a) and (b) 8.5, (c) and (d) 7 and (e) and (f) 6 under anoxic and oxic conditions.

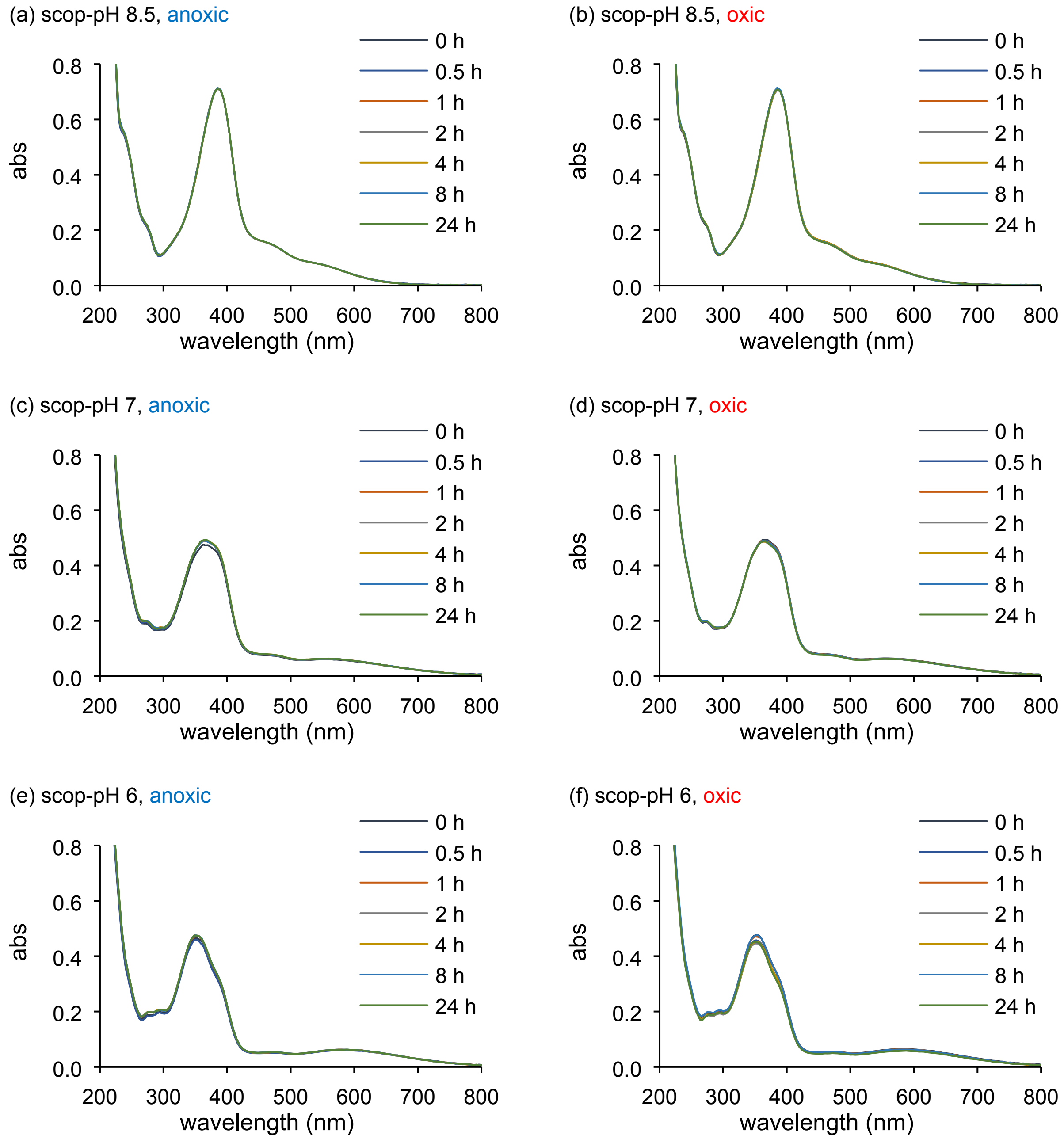

Figure S20. The change of UV–visible absorption (abs) spectra of Fe(III)-esc (10 μM Fe(III) and 42 μM esc) at pH (a) and (b) 8.5, (c) and (d) 7 and (e) and (f) 6 under anoxic and oxic conditions.

(a) esc-pH 8.5, anoxic

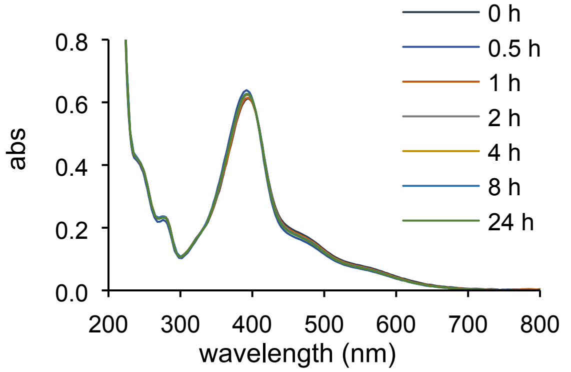

(b) esc-pH 8.5, oxic

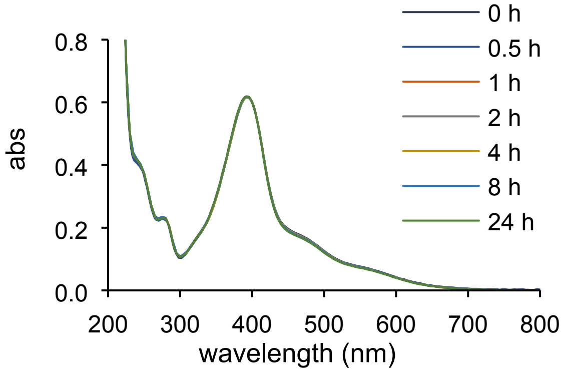

(c) esc-pH 7, anoxic

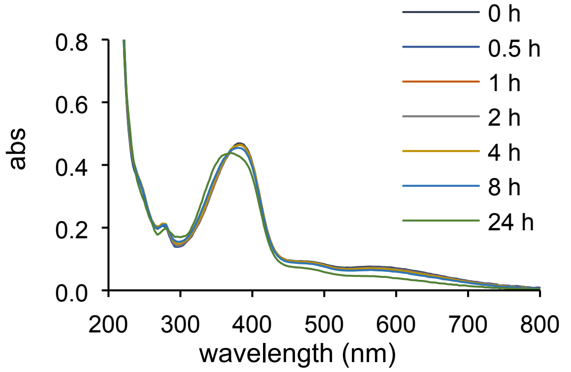

(d) esc-pH 7, oxic

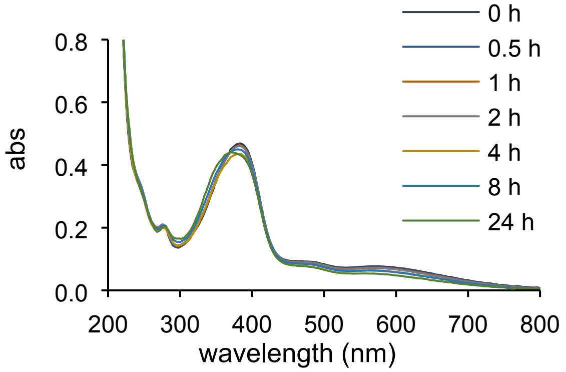

(e) esc-pH 6, anoxic

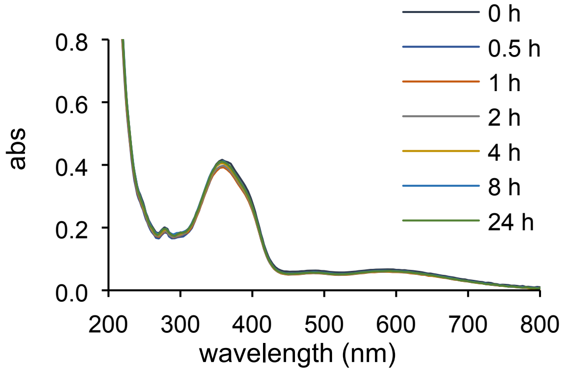

(f) esc-pH 6, oxic

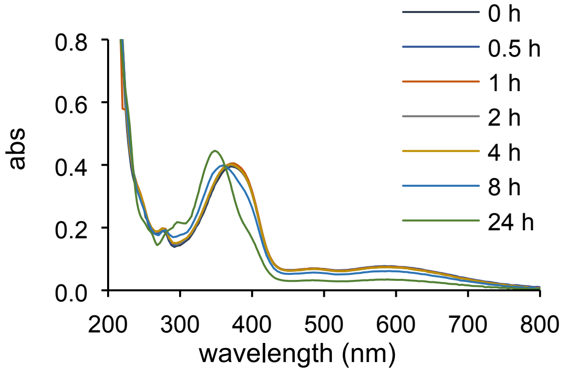

Figure S21. The UV–visible absorption (abs) spectra of (a) Ferrozine and Fe(III) + Ferrozine, (b) Fe(II)+Ferrozine and Fe(II)+Fe(III)+Ferrozine; and absorption spectra of Fe(II)/Fe(III)-coumarin (10 μM Fe(II)/Fe(III) and 83 μM of coumarin; (c-e) frax, (f-h) scop and (i-k) esc) in the presence/absence of 3 mM of Ferrozine under various pH conditions (pH 6 – 8.5). The spectra of Ferrozine, Fe(III) + Ferrozine, Fe(II) + Ferrozine and Fe(II) + Fe(III) Ferrozine were not changed between pH 6 to 8.5. For the Ferrozine treatment, the spectra have been analyzed immediately after the addition of Ferrozine to the Fe(II)/Fe(III)-coumarin solutions.

(a) Ferrozine and Fe(III)+Ferrozine

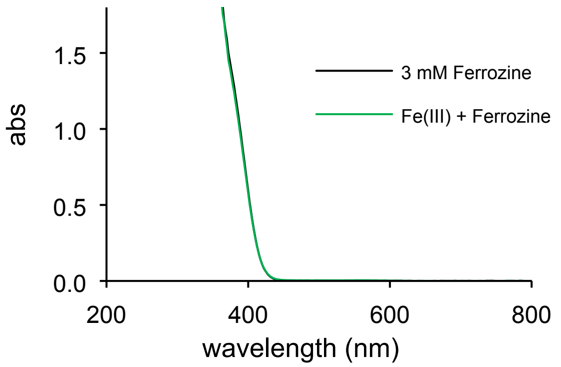

(b) Fe(II)+Ferrozine, Fe(II)+Fe(III)+Ferrozine

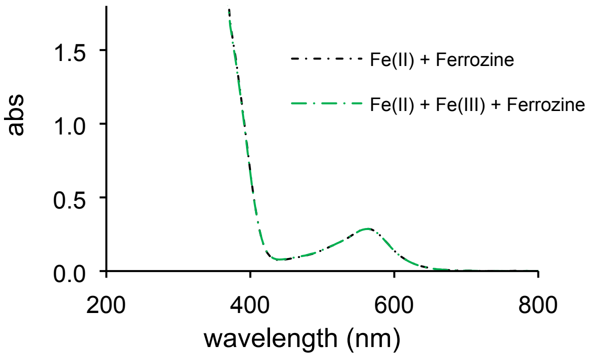

(c) frax, pH 6

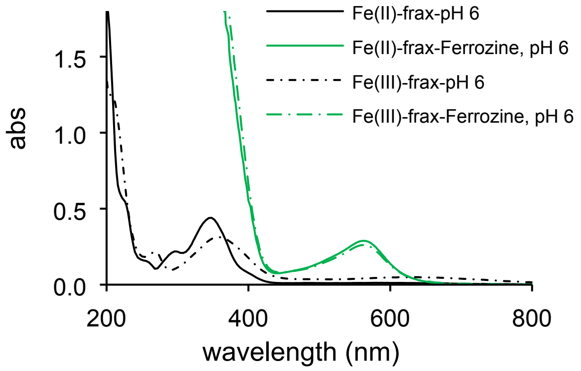

(d) frax, pH 7

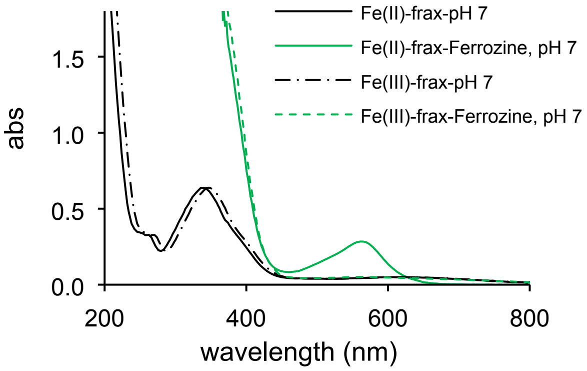

(e) frax, pH 8.5

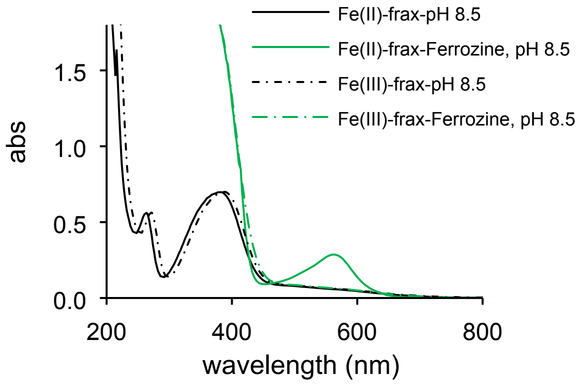

(f) scop, pH 6

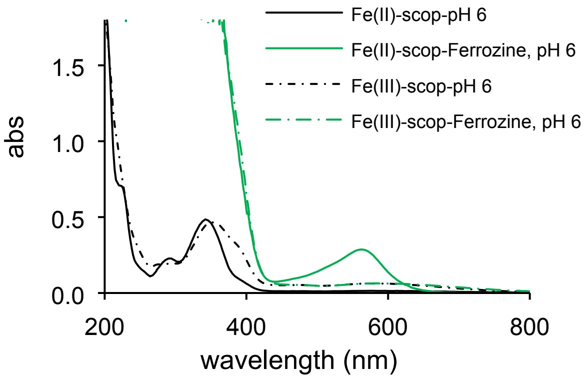

(g) scop, pH 7

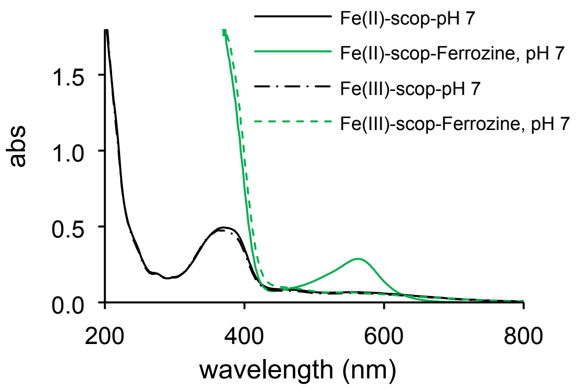

(h) scop, pH 8.5

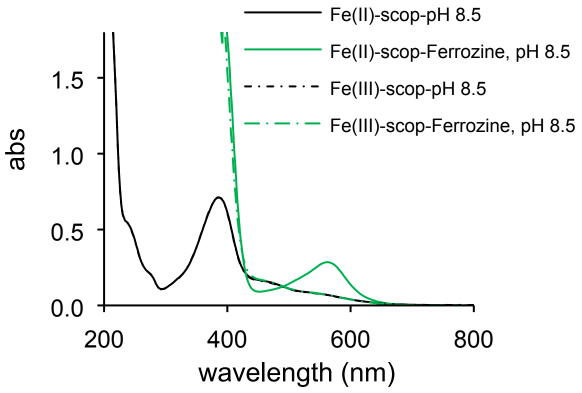

(i) esc, pH 6

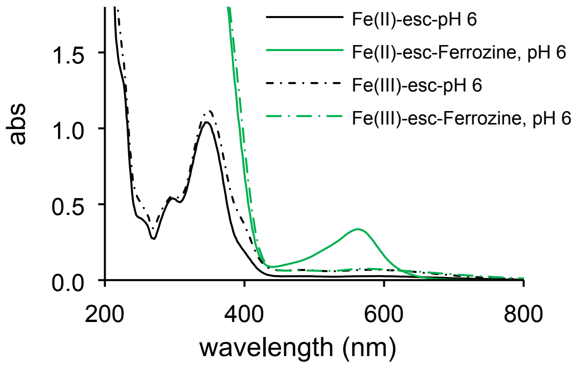

(j) esc, pH 7

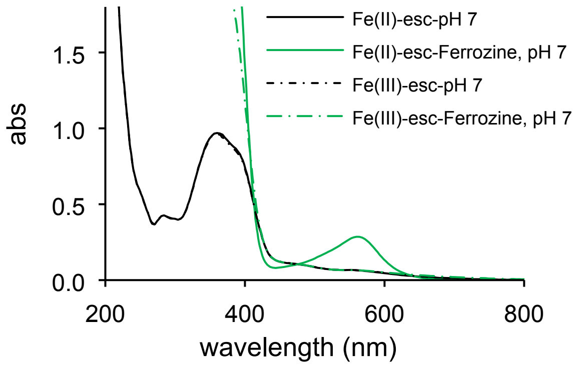

(k) esc, pH 8.5

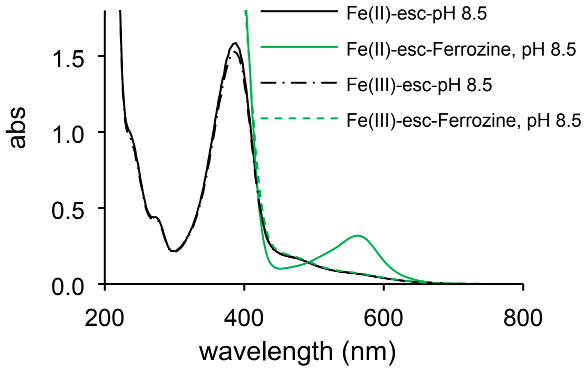

Figure S22. Linear relations between the concentration of Fe(III)-coumarin complexes in the presence of a stoichiometric excess of coumarin ligand (Fe(III): 0.5-20 μM and coumarin: 83 μM) and absorbance a 563 nm, for pH 6, 7 and 8.5

(a) Fe(III)-frax, pH 6

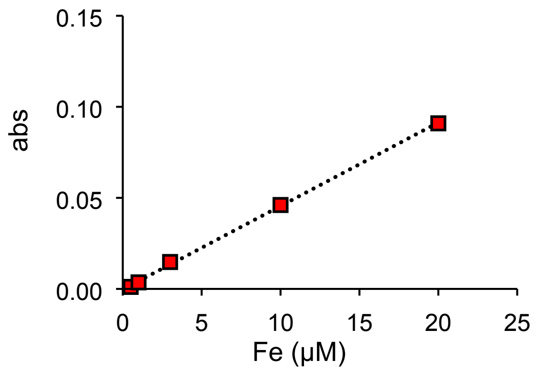

(b) Fe(III)-frax, pH 7

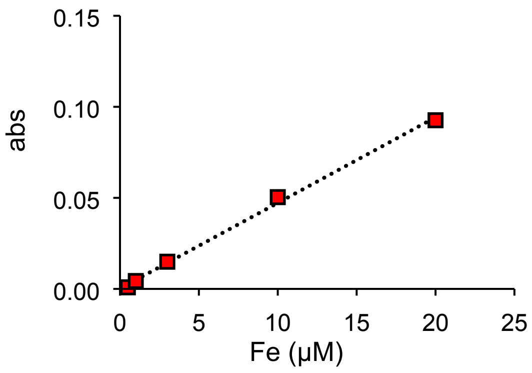

(c) Fe(III)-frax, pH 8.5

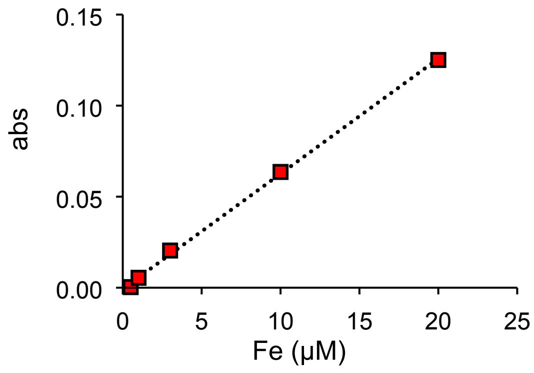

(d) Fe(III)-esc, pH 6

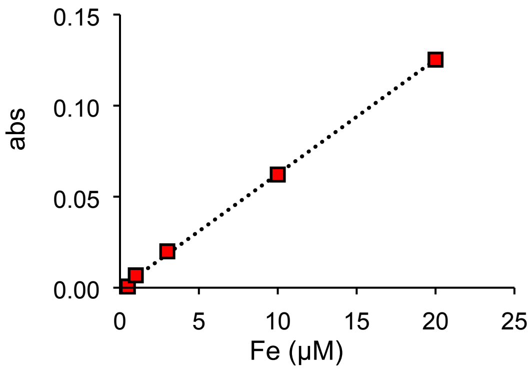

(e) Fe(III)-esc, pH 7

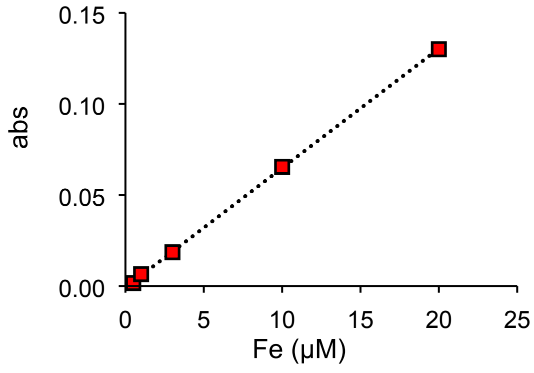

(f) Fe(III)-esc, pH 8.5

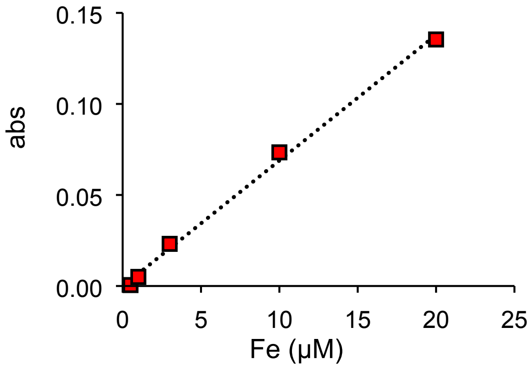

(g) Fe(III)-scop, pH 6

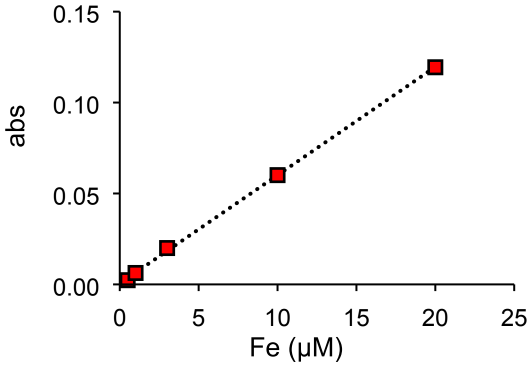

(h) Fe(III)-scop, pH 7

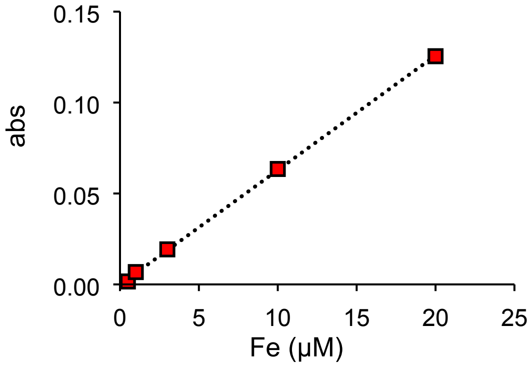

(i) Fe(III)-scop, pH 8.5

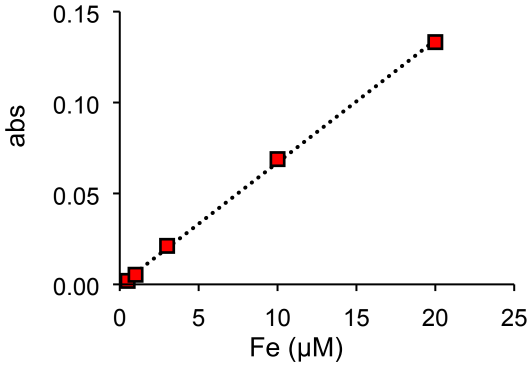

Figure S23. Change in UV–visible absorbance (abs) spectra of (a)-(c) Fe(III)-frax, (d)-(f) Fe(III)-scop and (g)-(i) Fe(III)-esc (10 μM Fe(III) and 83 μM frax, scop and esc) in the presence of 3 mM Ferrozine over time at pH 6, 7 and 8.5 under anoxic condition.

(a) pH 6, Fe(III)-frax, Ferrozine

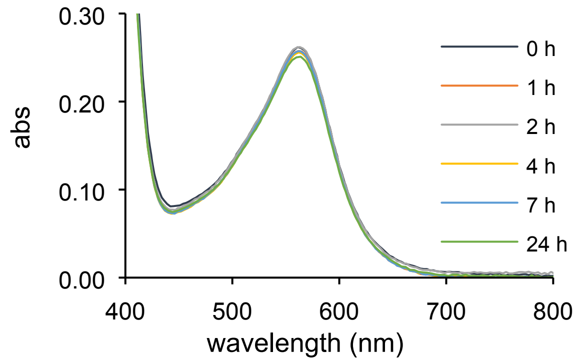

(b) pH 7, Fe(III)-frax, Ferrozine

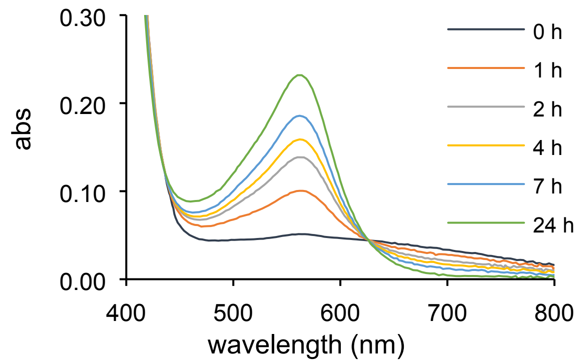

(c) pH 8.5, Fe(III)-frax, Ferrozine

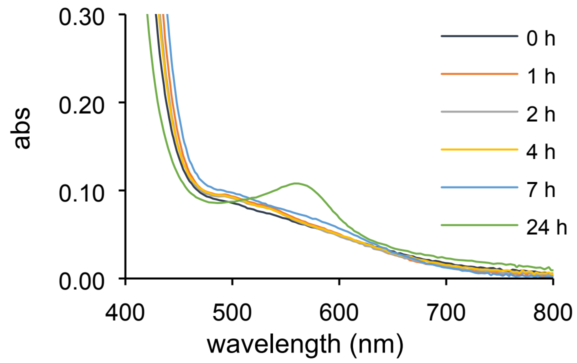

(d) pH 6, Fe(III)-scop, Ferrozine

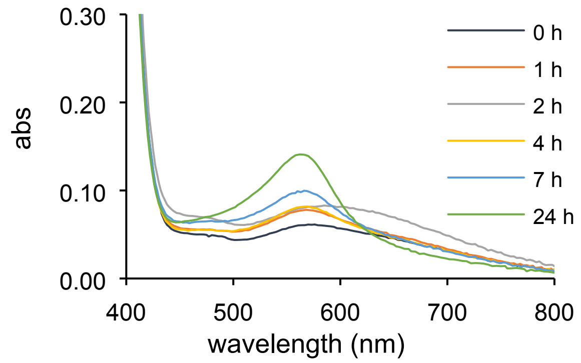

(e) pH 7, Fe(III)-scop, Ferrozine

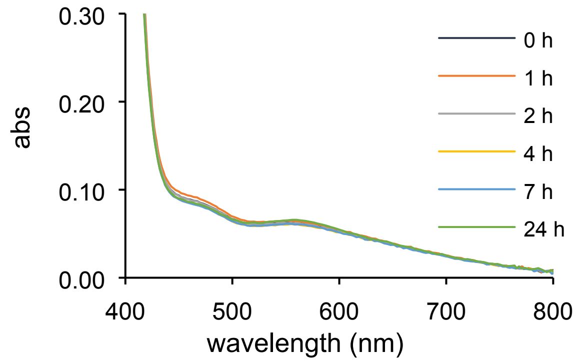

(f) pH 8.5, Fe(III)-scop, Ferrozine

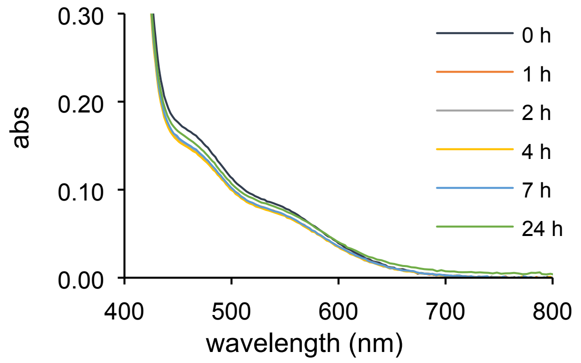

(g) pH 6, Fe(III)-esc, Ferrozine

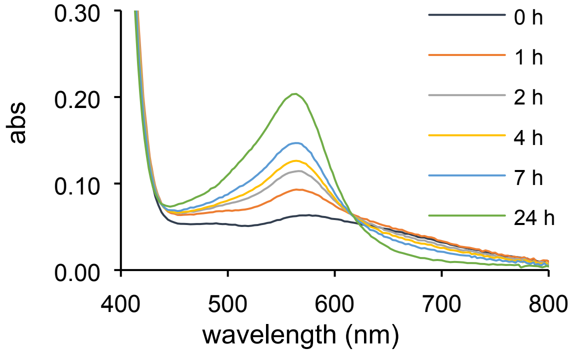

(h) pH 7, Fe(III)-esc, Ferrozine

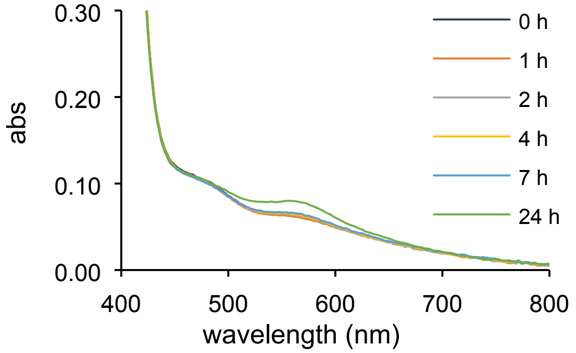

(i) pH 8.5, Fe(III)-esc, Ferrozine

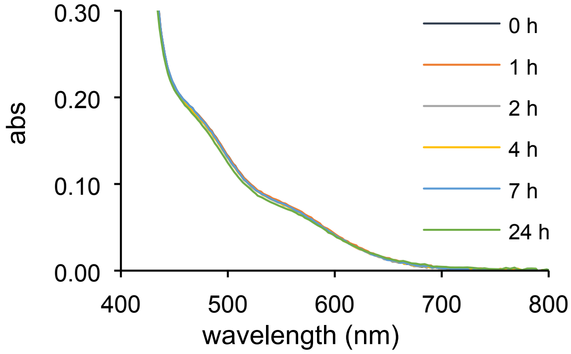

Figure S24. Change in UV–visible absorbance (abs) spectra of (a)-(c) Fe(III)-Fe(II)-frax, (d)-(f) Fe(III)-Fe(II)-scop and (g)-(i) Fe(III)-Fe(II)-esc (10 μM Fe(III), 10 μM Fe(II) and 83 μM frax, scop and esc) in the presence of 3 mM Ferrozine over time at pH 6, 7 and 8.5 under anoxic condition.

(a) pH 6, Fe(II)-Fe(III)-frax, Ferrozine

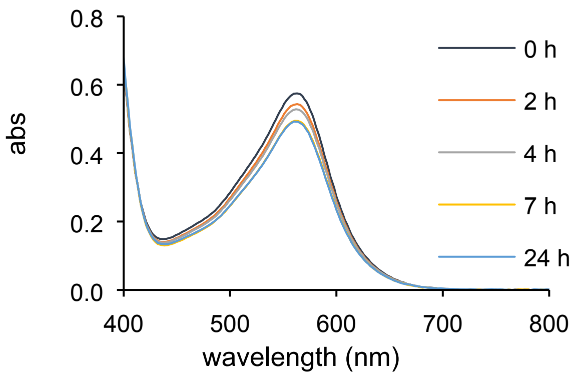

(b) pH 7, Fe(II)-Fe(III)-frax, Ferrozine

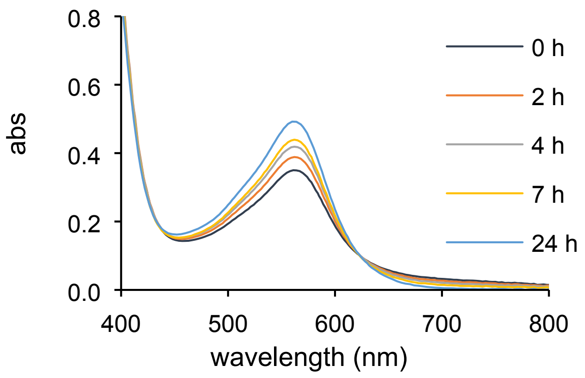

(c) pH 8.5, Fe(II)-Fe(III)-frax, Ferrozine

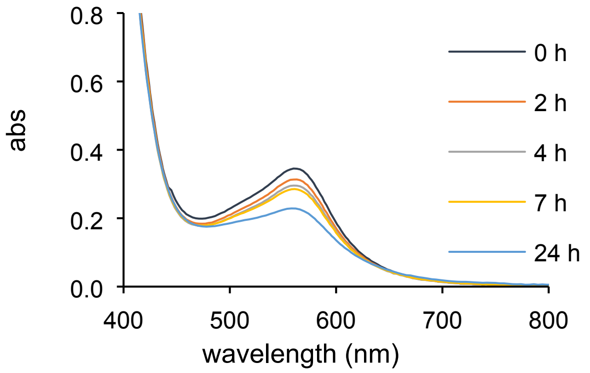

(d) pH 6, Fe(II)-Fe(III)-scop,Ferrozine

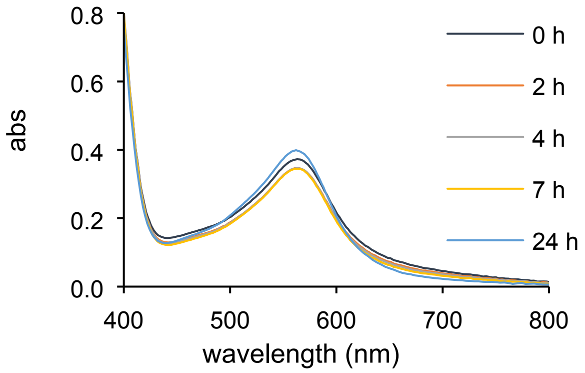

(e) pH 7, Fe(II)-Fe(III)-scop, Ferrozine

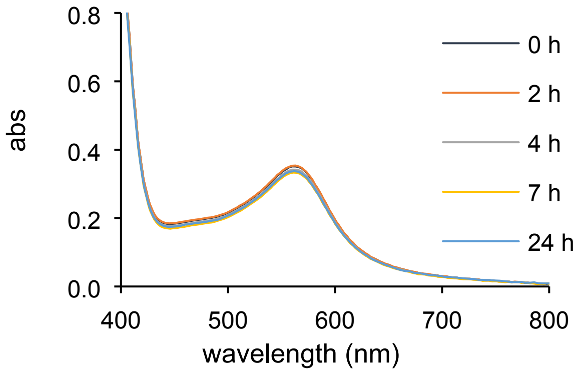

(f) pH 8.5, Fe(II)-Fe(III)-scop, Ferrozine

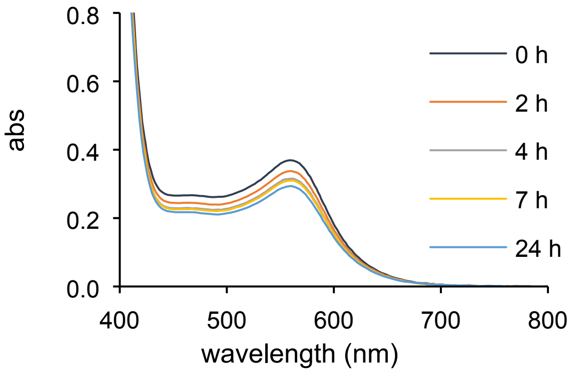

(g) pH 6, Fe(II)-Fe(III)-esc, Ferrozine

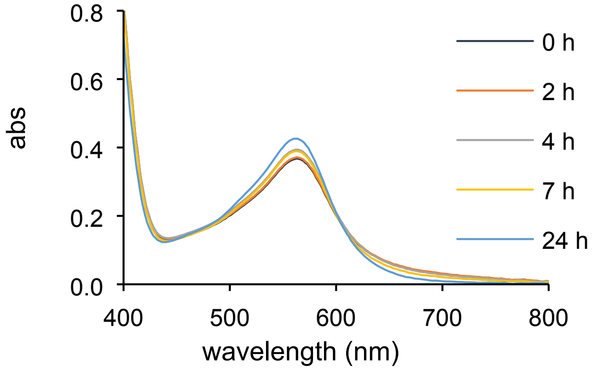

(h) pH 7, Fe(II)-Fe(III)-esc,Ferrozine

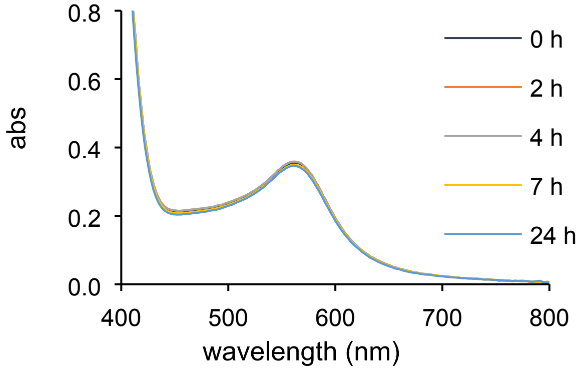

(i) pH 8.5, Fe(II)-Fe(III)-esc, Ferrozine

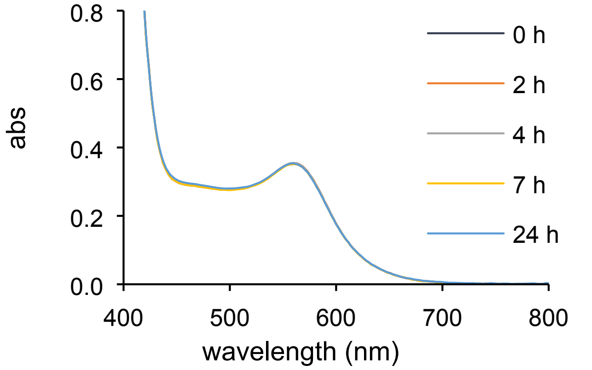

Figure S25. The changes in Fe speciation of (a)-(c) Fe(III)-Fe(II)-frax (d)-(f) Fe(III)-Fe(II)-scop and (g)-(i) Fe(III)-Fe(II)-esc (10 μM Fe(II), 10 μM Fe(III) and 83 μM coumarin) in the presence of 3 mM Ferrozine over time at pH 6, 7 and 8.5 under anoxic condition.

(a) Fe(III)-Fe(II)-frax

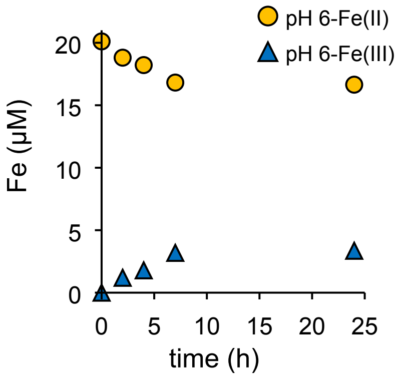

(b) Fe(III)-Fe(II)-frax

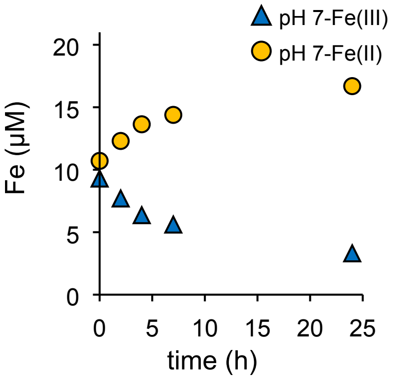

(c) Fe(III)-Fe(II)-frax

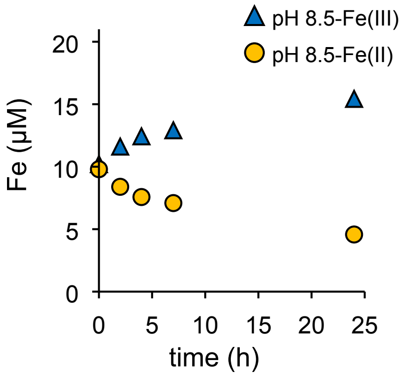

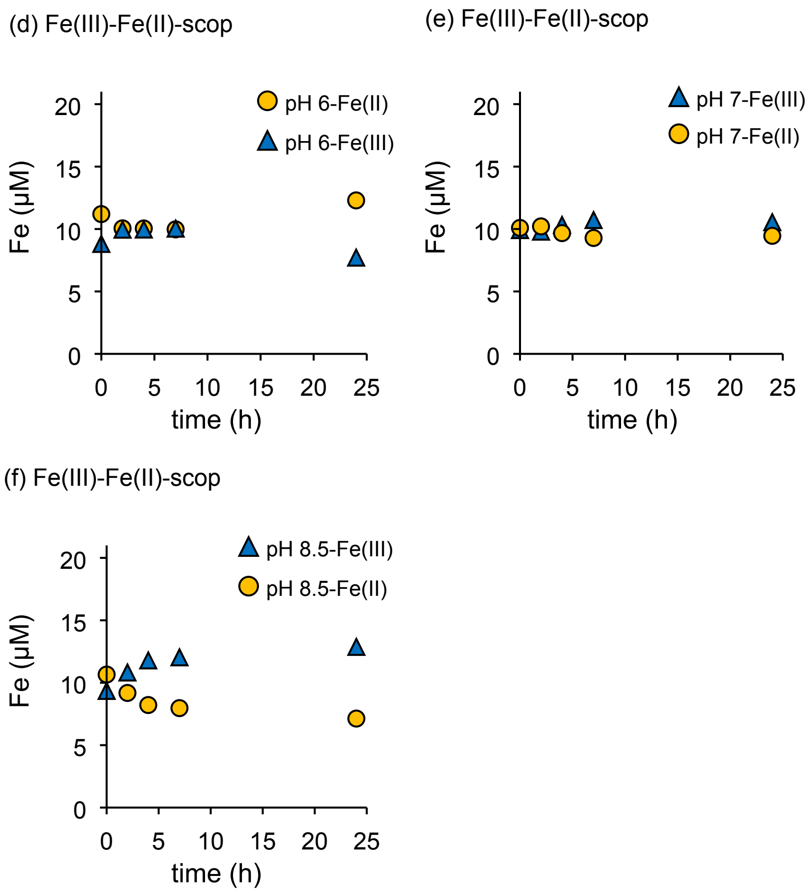
(d) Fe(III)-Fe(II)-scop
pH 6-Fe(II)
pH 6-Fe(III)
Fe (μM)
time (h)
(e) Fe(III)-Fe(II)-scop
pH 7-Fe(III)
pH 7-Fe(II)
Fe (μM)
time (h)
(f) Fe(III)-Fe(II)-scop
pH 8.5-Fe(III)
pH 8.5-Fe(II)
Fe (μM)
time (h)

(g) Fe(III)-Fe(II)-esc

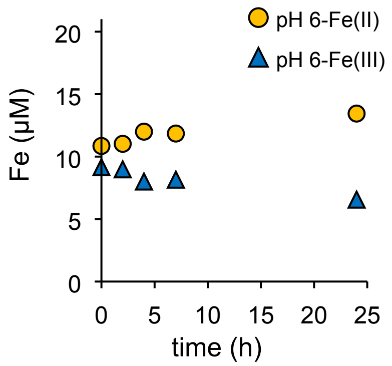

(h) Fe(III)-Fe(II)-esc

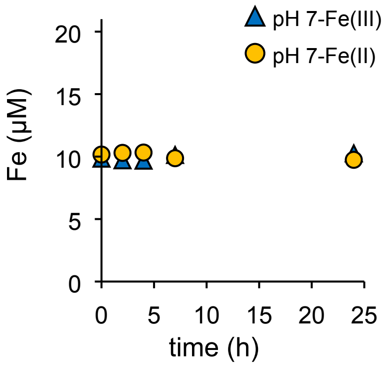

(i) Fe(III)-Fe(II)-esc

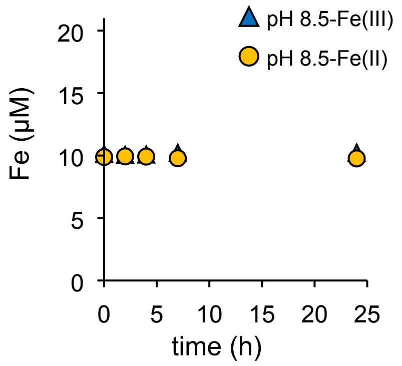

Figure S26. Procedure for calculating the initial Fe(III) reduction rates. Reduction rates were calculated from the slopes of linear regression lines of the Fe(II) concentrations; for treatments in which less than 50% of the Fe(III) was reduced in the course of the experiment, the entire time interval was included; for treatments in which more than 50% of the Fe(III) was reduced in 24h the regression was carried out over the first 4 h, because in these treatments the rate changed considerably during.

(a) Fe(III)-frax, pH 7

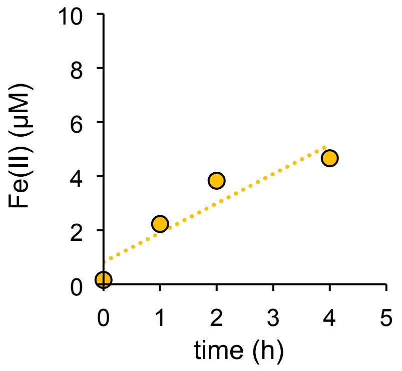

(b) Fe(III)-frax, pH 8.5

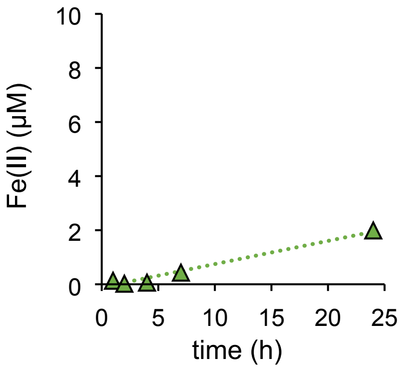

Table S3. Initial Fe(III) reduction rates based on the Fe concentration data in Figure 7 and Figure S23; negative rates represent oxidation.

pH	Fe(III)-frax (μM h^{-1})	Fe(III)-scop (μM h^{-1})	Fe(III)-esc (μM h^{-1})
6	538*	0.14	0.68
7	1.08	-	-
8.5	0.086	-	-
	Fe(III)-Fe(II)-frax (μM h^{-1})	Fe(III)-Fe(II)-scop (μM h^{-1})	Fe(III)-Fe(II)-esc (μM h^{-1})
6	600*	-	0.10
7	0.73	-	-
8.5	-0.56	-0.61	-

*The Fe(III) reduction rates in a mixtures containing Fe(III), fraxetin and Ferrozine at pH 6 were too large to estimate by regression; instead a minimum rate was estimated based on the change in Fe(III) concentration and the time (1 min) between Ferrozine addition and analysis.

Figure S27. Fe(II) recovery from Fe(III)-Fe(II)-esc (10 μM Fe(II), 10 μM Fe(III) and 83 μM esculetin) by 3 mM Ferrozine at pH 6, 7 and 8.5. The Fe(II)- esculetin and Fe(III)-esculetin solution were prepared either freshly or 1 day before under anoxic conditions.

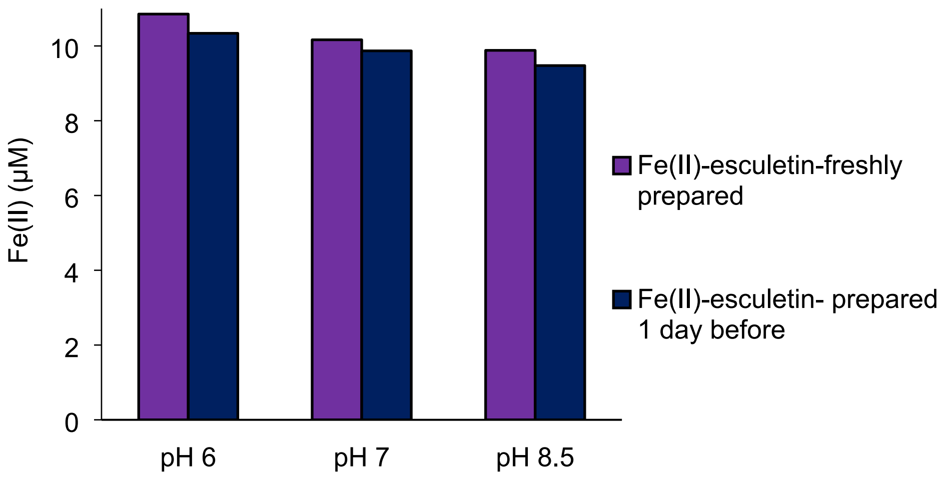

Figure S28. Electrochemistry of coumarins at pH 5. Cyclic voltammograms are shown of pure coumarin (blue colour) and after addition of Fe(III) (red colour). All measurements were done in anoxic solutions of 20 mM ammonium acetate at pH 5, using a glassy carbon working electrode (2 mm diameter), Ag/AgCl/KCl (3 M) reference electrode, and Pt counter electrode. The scan rate was 100 mV s^{-1}.

(a) 230 μM esculetin (blue), + 45 μM Fe(III) (red)

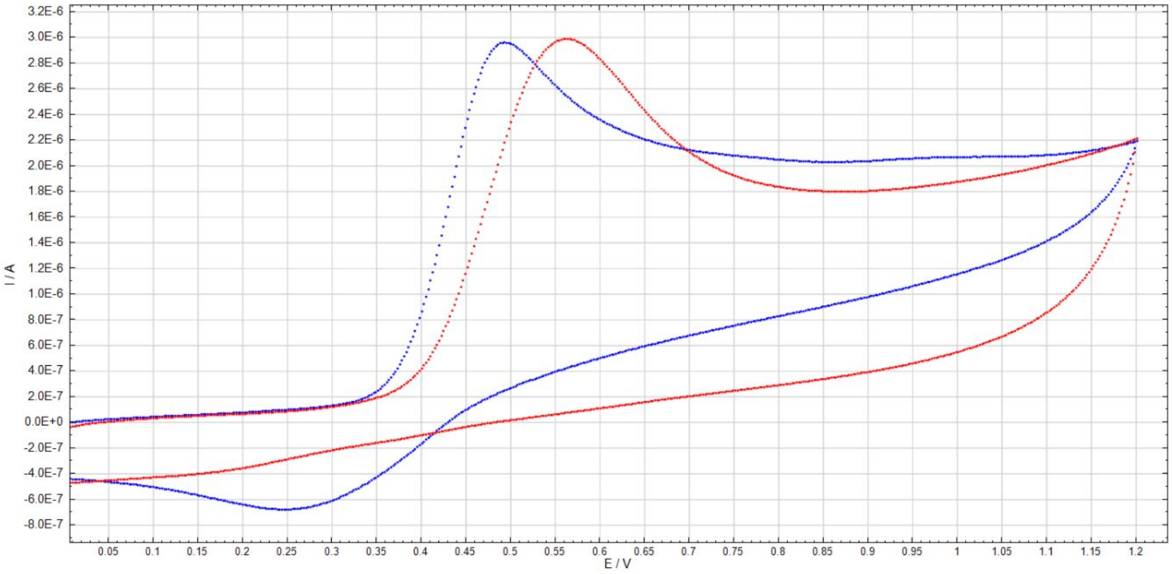

(b) 220 μM fraxetin (blue), + 45 μM Fe(III) (red)

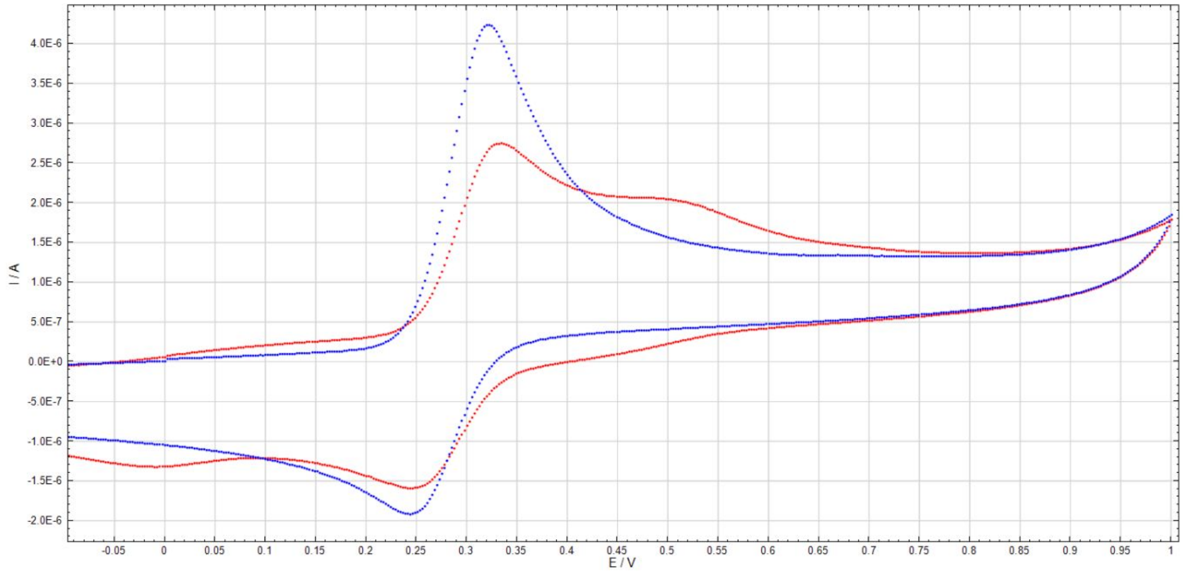

(c) 230 μM scopoletin (blue), + 43 μM Fe(III) (red)

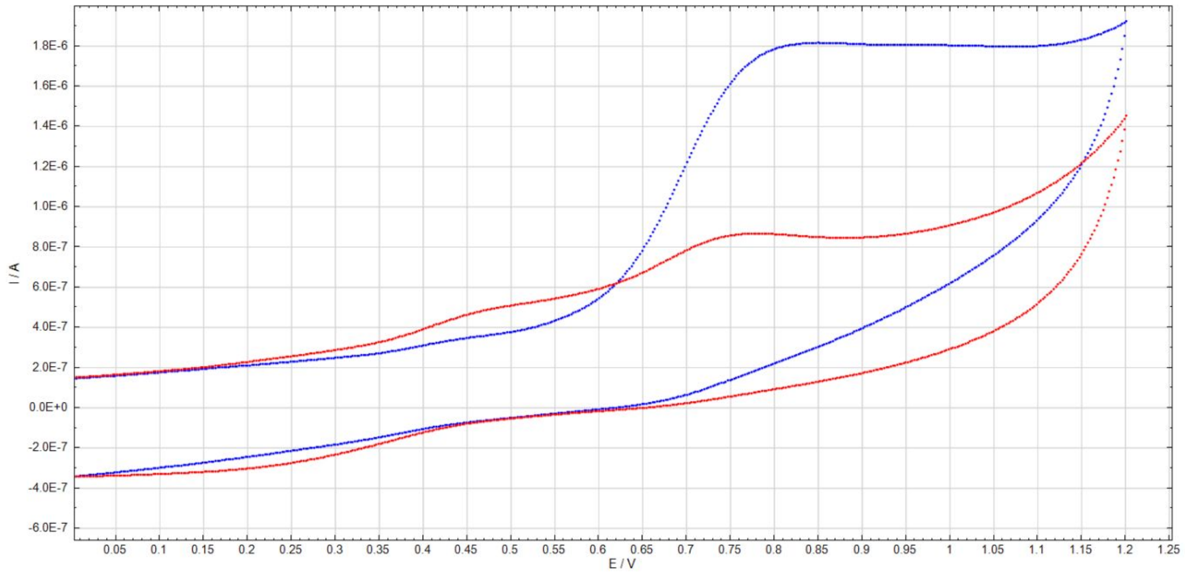

Supplement: Supplementary file 1 — sp3c00199_si_001.pdf [file sp3c00199_si_001.pdf]
